# What’s left after the hype? An empirical approach comparing the distributional properties of traditional and virtual currency exchange rates

**DOI:** 10.1371/journal.pone.0220070

**Published:** 2019-07-26

**Authors:** Alexander Hempfing

**Affiliations:** Bamberg Doctoral Research Group on Behavioral Macroeconomics (BaGBeM), University of Bamberg, Bamberg, Bavaria, Germany; Universidad Veracruzana, MEXICO

## Abstract

This paper provides an empirical analysis of the distributional properties and statistical regularities of virtual, intra-virtual and traditional currency exchange rates. To perform the analysis, the most relevant virtual, intra-virtual and foreign currency exchange rates between October 2015 and December 2018 are examined. The analysis shows that, in spite of their differing mode of formation, daily log-returns of all currency types share tent-shaped empirical densities, one of the characteristics of a Laplace distribution at semi-log scale. This peculiar property has also been examined thoroughly in other fields of economic literature. Moreover, the empirical results show that virtual and traditional currencies hold the same functional form, even after the 2018 hype. However, in spite of these similarities virtual and intra-virtual currencies display fatter tails and steeper towering peaks than regular foreign currencies which underscores the rather speculative nature of this asset class.

## Introduction

Money and innovation are central to capitalist economies. While money can be described as a medium of exchange, being fully liquid and used to make or receive payments for goods and services [1], innovation is more difficult to define. In its Oslo Manual, the Organisation for Economic Co-operation and Development (OECD) describes innovation as “the implementation of a new or significantly improved product (good or service), or process, a new marketing method, or a new organizational method, in-business practices, work-place organization or external relations” [2].

However, whereas the concept and technology of (fiat) money remained constant for decades, the concept of innovation has evolved tremendously; see, for example, [3] or [4]. Innovation reaches into all aspects of our lives. It was therefore only a matter of time before innovation began to affect our concept of money, and how we transfer and receive means of payment.

In 2009, Satoshi Nakamoto, an anonymous person or group, published a paper entitled “Bitcoin: A Peer-to-Peer Electronic Cash System”. The paper describes a concept that enables online payments to be made between two parties without the need for an intermediate entity, such as a financial institution. Instead, they propose a protocol, arranged as a decentralized network using a chain of hash-based proof-of-work, forming a record that cannot be forged. [5] demonstrates the system’s unforgeability using the example of a binominal random walk. Records cannot be forged because changing the information in the chain requires that the majority of nodes in the network approve this change and write it into a general ledger. Such a ledger is also referred to as a “Blockchain” [5].

Nevertheless, it is important to explore how virtual currencies such as Bitcoin, Etherium and Litecoin are formed, and how they compare to traditional money around the world. In traditional fiat money systems, the theory is that central banks cover for aspects such as price stability by controlling the interbank interest rate via open market operations; in contrast, most virtual currencies are “mined”. In the case of Bitcoin, for example, involving a competitive and decentralized process, specific hardware and software are used to solve cryptographic hash functions. In the process, a “hash” describes a hexadecimal number with a particular target difficulty that needs to be explored by the nodes in the system to create or solve for a new block [6]. Based on a predetermined schedule Miners who successfully participate in solving the block are rewarded a specific amount of Bitcoins, bound to their computational power [7].

Before exploring the key question of this paper, which is how virtual currencies are distributed and how they behave compared to traditional currencies, other matters of interest must first be addressed. First, why do virtual currencies, such as Bitcoin, have a value? What determines their price? How are they traded? Are virtual currencies a safe means of payment, and what are their disadvantages compared to traditional currencies? In addition, light must also be shed on the growing body of research into this phenomenon in recent years, which will be performed in the next section.

The main motivation for this paper is to investigate several virtual currencies, including Bitcoin, and to compare their statistical properties and regularities using a parsimonious continuous probability distribution. Another objective is to compare, how the stylized facts of real and virtual currency exchange rates differ, and whether they exhibit similarities. In an attempt to provide an extensive picture, an analysis is therefore undertaken of the four most significant exchange rates of actual to virtual currencies, three intra-virtual currency exchange rates and the four foreign currency exchange rates, including three major and one minor fiat currency.

The remainder of this paper is organized as follows: Public perception and rising academic interest in recent years introduces the academic and public debate, creating a reference to the current literature and answering the aforementioned question. Data and Descriptive Statistics describes the data and descriptive statistics. Empirical Framework introduces the empirical set-up, and outlines the method used to construct the empirical analysis. Afterwards Results contains the main results, setting their implications into relation to current findings. The paper concludes with a Discussion and Conclusion, also highlighting a number of limitations and ideas for future research.

Outlining the results briefly, it was possible to show that virtual, intra-virtual and foreign currency log-returns are tent-shaped one of the characteristics of a Laplace distribution at semi-log scale and share the same functional form. This peculiar property has also been well examined thoroughly in other fields of economic literature [8–10]. Furthermore, virtual and intra-virtual currencies exhibit higher volatility, fatter tails and steeper towering peaks than regular foreign currencies, while following the stylized facts of asset returns more extremely to some extent. It may therefore be better to consider virtual and intra-virtual currencies as a speculative instrument rather than an alternative to traditional currencies.

## Public perception and rising academic interest in recent years

Following the functional definitions of [11], any type of money is a medium of exchange, unit of account and store of value. [12] examine the defining characteristic of money, also including velocity, acceptability, and liquidity. In contrast to commodity money, which holds a value due to its physical properties, or fiat money, which is used as legal tender and mainly bases its value on trust in central authorities, virtual money is created by means of computational processes using a commonly predictable rate. Nonetheless, virtual money also bases its value on trust and the conviction that its concept is superior to other forms of currency.

Like other currencies, the price of virtual money is determined by supply and demand. To facilitate this process, it is traded through currency exchanges. However, this results in a number of limitations. Regulatory barriers are not only high for currency exchanges (they operate as “money transmitters” and must therefore be registered with the Financial Crimes Enforcement Network in the US, for example), they must also provide an online infrastructure that is sufficiently strong to withstand hacking attacks. The number of relevant high-volume exchanges is therefore small [13]. In December 2016, the three most significant exchanges (OKCoin, Huobi and BTC China) accounted for more than 97% of all (Bitcoin) trades over a six-month period [14].

However, the exchange provider market has experienced extreme fluctuations, especially over the last two years. By January 2018, the three largest exchanges had utterly changed (then bitfinex, coinbase, bitflyer) and accounted for a total market share of only 53% of all (Bitcoin) transactions over a six-month period [15]. By January 2019, after the hype, Bit-x and GDAX superseded coinbase and bitflyer. Still, the total market share of the top three providers remained unchanged [16]. Hence, the description provided in [13] is unlikely to remain valid, since the market for virtual currencies is growing tremendously, with more exchange providers competing for a piece of the cake.

Trading platforms such as Bitfinex are no different than typical trading platforms used to trade foreign currencies, stocks or futures. At Bitfinex, for example, it is possible to trade Bitcoin (BTC), Ethereum (ETH) and Litecoin (LTC) on a spot price, that describes a current price in the marketplace at which the currencies can be bought or sold for immediate delivery. In the process, the platform offers different display options, like candlesticks, ranging from one minute to one day. Also, conventional indicators such as the Moving Average Convergence/Divergence (MACD) or Relative Strength Index (RSI) can be used to identify or ease trading opportunities and decisions [17]. However, the authors of [13] point out that virtual currencies mirror a payment platform rather than what economists consider a currency, because Bitcoin exchange refers to a fixed amount of a conventional currency. In another economic appraisal, [18] concludes that Bitcoin might function somewhat like a speculative investment rather than a currency. In contrast, [19] find from a wavelet coherence analysis that standard fundamental factors—usage in trade, money supply and price level—play a role in Bitcoin price in the long term, which is generally in line with monetary economics theory.

Nonetheless, safety plays a vigilant role in trading and dealing with virtual currencies. When [5] proposed his electronic cash system, he argued that, due to the expansion of online commerce, financial institutions deprave to trusted third parties handling electronic payment processes, while continuing to inherit the weaknesses of being based on the trust model. It is not possible for financial institutions to reverse a transaction, such as when an individual is not liquid or when an operation is canceled without additional costs. These additional costs even increase in the case of non-reversible payments of non-reversible services. Therefore, [5] recommends, “an electronic payment system based on cryptographic proof instead of trust, allowing any two willing parties to transact directly with each other without the need for a trusted third party”. Following this line of argumentation, this system would protect sellers from fraud because it would be computationally impractical to reverse transactions, and buyers would be safeguarded through routine escrow mechanisms. Thus, in theory, as a means of payment, the system would be a suitable alternative to traditional methods of payment.

However, the design of virtual currencies, such as Bitcoin, exhibits characteristic risks that vary compared to other currencies or methods of payment. In a review paper, [13] focus on aspects such as market, counterparty, transaction, operational, legal and regulatory risks. They argue that the sharp movements within the exchange rate (USD/BTC) between 2013 and 2015 could serve as a source of concern when being used for transactions or store of value. [20] finds that counterparty risk also has a virtue influence, when contemplating virtual currency exchanges. In their study, they conclude that, out of 40 surveyed exchanges, 18 closed after a median lifetime of 381 days, five of which failed to reimburse customers who held currencies on their accounts. Hence, loss of funds is a considerable risk. The most well-know case was Mt. Gox, which went bankrupt in 2014.

Moreover, due to the growing interest in virtual currencies, the costs of transacting virtual currencies among wallets or the general trade fee on trading platforms can also become a significant bottleneck A wallet is a software program where virtual coins can be stored. Wallets facilitate the sending and receiving of virtual coins, and give ownership of the balance to the user. With transaction, deposit/withdrawal and trading fees of up to 5% of the order value per operation, one could talk of predatory pricing behavior. In January 2018, for example, the average Bitcoin transaction fee was 28 USD; in mid-December 2017 it was 55 USD per transaction. By January 2019, the transaction fee dropped to 20 USD cents per execution [21]. Finally, another risk related to the transaction of virtual currencies is time. Although the average seven-day transaction time (January 2019) is 15 minutes to execute an order, this can vary widely. In early 2018, for example, transactions took an average of 2,521 minutes (almost two days) to settle [22].

Furthermore, besides transaction risk, operational risk must also be considered. In an article, [23] describe the risk of a history-revision attack. The authors describe the case where, if any party in mining were able to gain more than 50% of the computational power in the decentralized network mining the coins, the entire coin base could be replaced by a figment of its forgery. This, however, seems to be rather difficult to achieve. In December 2016, the largest mining pool “AntPool” had a mining market share of 19% [24]. Throughout the turbulent year of 2017, this did not change [25]. By 2019 however, AntPool was ousted by BTC.com, with a mining capacity of the total mining market of 15.4% [22]. It is important to note a concentration among the country of origin of the different mining pools: 81% of these are based in China [26]. Nevertheless, the scientific community is aware of these security issues and challenges [27] and is searching for solutions, for example, by means of quantum computation which would improve the level of security by the laws of physics, a state not achievable from a non-quantum information theoretic viewpoint [28].

Finally, Bitcoin and other virtual currencies are subject to legal and regulatory risks. [13] therefore raise concerns in the field of (financial) crime (for example, money laundering) and consumer protection. However, there is a more vigilant concern: politics and monetary policy regulation. In an opinion of the European Central Bank (ECB) as early as in 2016, the authors state that “[…]the reliance of economic actors on virtual currency units, if substantially increased in the future, could in principle affect the central banks’ control over the supply of money with potential risks to price stability[…]” [29]. Also, in 2017, the ECB continued to warn about the dangers of investing in digital currencies. In September, Vice President Vitor Constancio compared the Bitcoin hype with the 17th century tulip mania [30]. Benoît Cœuré, Member of the Executive Board of the ECB, argued: “Bitcoin is not a currency; it is a financial instrument which creates major risks for investors because its value is highly unstable, […]” [31]. Benoît Cœuré was proven correct after the Bitcoin price dropped from around 20,000 USD in early 2018 to under 5,500 USD by the end of the year, dubbing it in an interview with the Financial Times “an evil spawn of the financial crisis” [32]. Similar views were shared by Augustin Carstens, General Manager of the Bank for International Settlements (BIS) [33].

Yanis Varoufakis, Greek economist and Greece’s former Minister of Finance, warned in an article about decentralized and “apoliticized” money such as Bitcoin, arguing that the only way to steer a course between Ponzi growth and stagnation is to exercise a degree of rational, collective control over the supply of money. Since such control is bound to be political, because different monetary policy decisions affect various groups of people, the only way to guarantee money for the people by the people is through a democratically controlled and collective agency [34]. Another strong argument is the deliberate choice of countries or states to devalue or depreciate their national currencies against foreign currencies. By devaluating their currency, countries can increase their market competitiveness in an open economy, while inheriting the currency purchasing power over some time in their own country. This approach would not be possible for countries that participate in a decentralized network, that renounce national control over their own monetary policy. This does not apply for countries within the Euro area, the monetary union of 19 of the 28 European Union Member States. Hence, it is understandable that regulatory institutions and the public are becoming increasingly sensitive to the subject of virtual currencies, their properties and effects on the economy. Not surprisingly, academic research in this field has gathered pace in recent years, too.

Discussing the economics of Bitcoin, [35] analyze the effect of volatility on prices using autoregressive conditional heteroscedasticity (ARCH) and generalized autoregressive conditional heteroscedasticity (GARCH) models. The authors find that the effect is significant. [36] investigate whether Bitcoin could serve as a financial asset to diversify a investor’s portfolio. Weighing up the risks they found it to be an attractive opportunity for the future. In [37] the authors analyze Bitcoin transactions, and find that sublinear preferential attachment governs the evolution of wealth distribution within the transaction network. Examining the relationship between several virtual currencies, looking at how network effects influence the cryptocurrency market, Gandal and co-authors conclude that there may be statistical arbitrage opportunities due to comovement among several of these currencies [38].

However, little attention has been paid to the statistical properties of virtual currencies; and how they compare to traditional currencies. While [39] conduct a risk assessment for Bitcoin and its extreme tail behavior, [40] looks beyond Bitcoin and examines the statistics of other virtual currencies, too, finding that they all exhibit heavy tails. Similar methods are applied by [41], fitting fifteen of the most popular parametric distributions in finance to the log-returns of Bitcoin. They find that the Generalized Hyperbolic Distribution (GH) fits the empirical distribution of Bitcoin best.

Still, there are some constraints to these results. The five parameters required to estimate the GH are not parsimonious in an economic sense. Also, as a general form, it builds a superclass of several distributions, including the Student’s t-Distribution, the Laplace, and Hyperbolic distribution, which makes it harder to distinguish the origins of its distributional properties. Moreover, the variety of parameters makes it difficult to interpret the underlying basis from an economic perspective. Due to its semi-heavy tails and the variety of parameters, the GH is often used to model financial markets and such like. Nevertheless, narrower distributions with fewer parameters may be of greater interest. Thus, it is interesting to read that the Laplace distribution and the Exponential Power or Subbotin distribution were the best fitting distributions, with two and three parameters, respectively [41].

Moreover, applying (asymmetric) Laplace distributions to financial data, especially to exchange rate returns, has proven to be an effective solution. [42] fit (asymmetric) Laplace laws to daily log exchange rate returns, finding that they reflect properties of empirical data much better than other two-parametric distributions. Additionally, due to their one-dimensional and multivariate densities, which have convenient computational forms, estimation procedures are practical and more comfortable to implement. [43] also provide examples of this being true for other fields in finance such as stock market returns, option pricing and value-at-risk models. Moreover, the peculiar properties of the Laplace distribution are also examined in detail in other fields of economic literature, see [8–10, 44] for example.

Furthermore, to compare the properties of exchange rate returns of traditional, foreign currencies and of virtual and intra-virtual currencies, it may be insightful to compare the stylized facts of logarithmic returns. Across a wide range of different speculative markets, certain universal properties have been found in recent years. These include uncorrelated raw returns, and an alternation of periods of low volatility with periods of high volatility. The former can be recognized by the fact that the autocorrelation function of raw returns tends to be significantly different to zero for all time lags, the latter by the fact that the autocorrelation function of absolute returns is positive and decays slowly. Hence one could talk of a long-range dependence or a long memory effect. These stylized facts, and others, are discussed in detail in [45–50].

## Data and descriptive statistics

The data can be clustered into four groups. In the first group, we find the four largest virtual currencies in recent years, valued against the US Dollar (USD). These are Bitcoin (BTC), Etherium (ETH), Ripple (XRP) and Litecoin (LTC). The hard forks Bitcoin Cash (BCH) and Etherium Classic (ETC) are disregarded. A hard fork is a radical change to the protocol that makes previous transactions valid (or vice versa); it constitutes a permanent divergence from the previous version of the blockchain. The second group consists of virtual currencies valued against the largest virtual currency on the market, BTC. In this group, we find ETH, XRP and LTC again. The third group includes the four foreign exchange rates valued against the Euro (EUR). These are the US Dollar USD, the British Pound Sterling (GBP) and the Japanese Yen (JPY). Moreover, the Turkish Lira (TRY) is examined as an example for a more volatile currency in recent years. Finally, the fourth group contains the above currency groups, but pooled into one sample. However, it is important to note that these sets have been standardized by subtracting the mean and dividing by the standard deviation, which leads to the mean being equal to zero and sigma to unity. This corrects for multi-modality in the pooled datasets.

Since virtual currencies and their trading on exchanges are relatively new, it is a cumbersome task to find the right data and frequencies, and compare them against traditional currencies over a longer period of time. To establish frequency comparability between all three currency groups, daily log-returns have been used, as traditional currency exchange rates are not free available on an intra-day level. Moreover, statistically it is an important factor to use same frequencies when trying to determine regularities between datasets. As show in [51], e.g. regressing two time series and considering two different frequencies (e.g. daily and weekly log-returns), it is shown than the variance of coefficients is approximately five times smaller using daily than weekly-log returns. Aggregational gaussianity is another factor. Increasing the time scale of Δt over which returns are calculated, the distribution tends to look like a normal distribution. This has also been proven for foreign currencies by [52]. Therefore, comparing the shape of distributions on different time scales needs to be viewed with caution [53].

However, as intra-day data are available for virtual currencies the statistical and distributional analysis in this paper is also conducted for the four major virtual currencies with an exchange rate price frequency of four hours. The results can be found in the Supporting information, as the focus of this paper lies on daily returns. Nevertheless, the results will be referenced in the according upcoming sections. Both data for virtual currencies valued against USD and the intra-virtual exchange rates valued against BTC were retrieved from poloniex.com for daily exchange rates between October 1, 2015 and December 31, 2018. Poloniex was chosen because it has a complete API database for several virtual currencies [54]. The same time period applies to intra-day virtual currencies. Since the ECB does not release reference rates on weekends or public holidays the time period for traditional currency pairs was extended to April 1, 2014 and December 31, 2018, so as to gain a comparatively large sample set. Euro foreign exchange reference rates were retrieved from the European Central Bank website in an XML hyperlink format.

Providing an overview of the virtual currency market, Table 1 contains information about the four exchange rates under consideration. The information was retrieved from [55]. As can be seen, Bitcoin achieves by far the largest market capitalization and trade volume, followed by Ethereum. It is intriguing to see how the virtual currency market has changed tremendously within the space of just one year. S1 and S2 Tables, retrieved from [56] to a earlier point in time, provide the same data for January 2017 and January 2018, where the market capitalization of Bitcoin increased 15 times and the capitalization of Etherium even 136 times, before dropping to the level displayed below.

**Table 1:** Market capitalization and trade volume information for selected virtual currencies.

Currency	Market Cap	Volume (24h)	Available Supply	Maximum Supply
Bitcoin	$62,826,332,646	$5,149,012,954	17,495,200 BTC	21,000,000 BTC
Ethereum	$12,367,840,710	$2,508,432,990	104,509,749 ETH	—
Ripple	$13,064,861,972	$415,643,519	41,040,405,095 XRP	100,000,000,000 XRP
Litecoin	$1,896,529,537	$572,027,104	60,131,350 LTC	84,000,000 LTC

The development of virtual currency market shares is also extraordinary. In January 2017, Bitcoin accounted for 85.3% of the Top 100 virtual currencies with the four currencies accounting for 93.1% of the market [56]. By January 2018, the market share of Bitcoin had shrunk to 34%, and the four aforementioned currencies accounted for a market share of only 66%. By January 2019, Bitcoin had recovered to 54%, with the four currencies accounting for 78%.

S1 Fig shows the price time series of the virtual currencies in group one. The hype around virtual currencies for all four currency pairs, which started in early 2017 and ended abruptly as the bubble burst in the second quarter of 2018, is clearly visible. The price time series for the second group of virtual currencies valued against BTC are shown in S2 Fig. It is apparent that the price is a fraction of the Bitcoin, and that the hype is less distinct than in the first group.

To enable a comparison of virtual and actual foreign currency distributions, the four currency pairs valued against the Euro (USD, GBP, JYP, TRY) were evaluated in the third group. S3 Fig shows the development of the foreign exchange rate price times series accordingly. In contrast to the first two groups, the price development in group three is less erratic; even though a trend behavior is visible, it does not constitute an exponential-like explosion, as is the case with virtual currency prices. An exception is the Turkish Lira, due to economical and political up- and downswings, which thus is an interesting candidate to compare.

In general, one limitation should be noted. The worldwide foreign exchange market is the largest financial market in the world. According to the Triennial Central Bank Survey of [57], the “turnover in global foreign exchange (FX) markets averaged $5.1 trillion per day in 2016”. Thus, this should not be neglected when comparing both markets. Even though the virtual currency exchange market grew considerably last year, it is by far incomparable in size. When dividing the total market capitalization of the whole virtual currency market today by turnover in global foreign exchange markets, it corresponds to less than 8% of traditional foreign exchange turnovers, for one day in April.

Following the stylized facts in [53], a brief look must also be taken of the descriptive statistics of different exchange rates. As can be seen in Table 2, on average, the USD declined against all virtual currencies over the sample period (the mean change is positive). This is also true for intra-day virtual currencies, as can be seen in S7 Table. A mixed picture is revealed for the intra-virtual and foreign exchange rates. Virtual currencies may therefore prevail in this picture, although this might be due to the vast number of market entries in previous years, which was not experienced in the traditional foreign exchange market.

**Table 2:** Descriptive statistics of virtual, intra-virtual and foreign exchange rates.

Currency	N	Mean	SD	Median	Skewness	Kurtosis
USD/BTC	1209	0.00222	0.04115	0.00316	-0.23246	6.75165
USD/LTC	1209	0.00195	0.05974	-0.00061	1.47794	16.0588
USD/ETH	1209	0.00427	0.06987	0.00003	0.39261	7.4049
USD/XRP	1209	0.00335	0.08162	-0.00134	2.42715	34.8900
BTC/LTC	1209	-0.00029	0.04655	-0.00321	3.08048	33.3706
BTC/ETH	1209	0.00202	0.06042	-0.00287	0.68773	7.62495
BTC/XRP	1209	0.00113	0.07697	-0.00390	2.86402	42.6591
EUR/USD	1215	-0.00015	0.00534	-0.00009	-0.22685	6.91474
EUR/GBP	1215	0.00006	0.00539	-0.00007	0.91837	12.3740
EUR/JPY	1215	-0.00010	0.00587	0.	-1.04531	14.2434
EUR/TRY	1215	0.0005	0.01014	0.00010	2.20288	34.0457
Pooled Virtual Currencies	4836	−1.26 ⋅ 10^−17^	0.99969	-0.04111	1.01631	16.2763
Pooled Intra-Virtual Currencies	3627	6.36 ⋅ 10^−18^	0.99972	-0.06758	2.21075	27.8849
Pooled Foreign Currencies	4860	1.46 ⋅ 10^−17^	0.99969	-0.01220	0.46227	16.8945

Variables are log-returns of the respective currencies. Pooled Exchange Rates are standardized for mean zero and standard deviation one.

Looking at the standard deviation, it is striking that both virtual currency groups experience a much higher standard deviation than foreign currencies. Such volatility, which, by implication, is higher is also apparent when volatility clustering is considered in the different datasets. This is visualized in S4, S5 and S6 Figs. It can be seen from these figures that virtual and intra-virtual log-returns exhibit much higher distortions than traditional currencies, while the clustering itself is more diffuse, too. Moreover, it is interesting to note that both volatility clustering and its intensity gathered pace over time. For example, the exchange rate returns of USD/LTC and BTC/LTC increased immediately after the hype around virtual currencies in early 2017. Nevertheless, actual currencies also display volatility clustering over time, albeit in a more sequential fashion.

The skewness of the exchange rate log-returns yields an inconsistent picture across classes. The log-returns for USD/BTC have a slightly negative (left) skew, which tends to be more pronounced than for other currencies; in contrast, BTC/XRP exchange rate log-returns experience an extreme positive (right) skew, outweighing all other pairs. All currencies are leptokurtic, i.e. they have positive excess kurtosis, which is more peaked and fat-tailed than the Gaussian distribution. Similar results were provided by [52] back in the late 1980s for foreign exchange rate returns and by [39] for virtual currencies in recent years. When increasing the frequency, the descriptive statistics in S7 Table reveal that the kurtosis increases significantly, which indicates high intra-day dynamics. Comparing daily virtual, intra-virtual and foreign currencies, the kurtosis of the first two types of currency is more extreme than that of the latter type. This becomes increasingly apparent when the currencies are pooled.

## Empirical Framework

To receive logarithmic exchange rate returns, let *P*_*i*_ be the closing price of a virtual, intra-virtual or foreign currency at time *i*. Hence, a return in one period can be defined as the relative change of *P* between *j* and *i*, where *j* = *i* − 1. Thus the simple net return is given by Eq (1)

$$R_{i}=\frac{P_{i}-P_{i-1}}{P_{i-1}}=\%\Delta P_{i}.$$ (1)

Following this approach, we can define continuously compound daily returns, *r*_*i*_ as Eq (2)

$$r_{i}=ln(\frac{P_{i}}{P_{j}})=ln\left(P_{i}\right)-ln\left(P_{j}\right),$$ (2)

where *r*_*i*_ can be called the log return. There are several advantages of using log returns, namely log-normality, approximate raw-log equality and time-additivity. These advantages result in the simplicity of multi-period returns, amongst others [51].

Building on the theoretical findings described above, the two distributions adduced to compare the distributional properties of traditional and virtual currency exchange rates are the Laplace distribution and the Exponential Power, or Subbotin, distribution. These distributions which are fitted against the virtual, intra-virtual and foreign currencies in this paper, are frequently used in finance; see [58] for the Subbotin distribution, for example. To the Laplace distribution an important economist already drew attention in the early 20th century. In his article entitled “The principal averages and the laws of error which lead to them”, [59] focused on the Laplace distribution, emphasizing the importance it gives to the median of sample errors. This was also supported by [60] in his survey of interest rates. It therefore, comes as no surprise that the Laplace distribution also plays a vigilant role in the attempt to find the best fitting distribution for data. [43] states that “an area where the Laplace and related distributions can find most interesting and successful applications is modeling of financial data”, also noting that they can be successfully applied to changes in currency exchange rate [43].

Briefly introducing the relevant distributions, let the probability density function (PDF) of *r* be represented as *f*(*r*). Consequently, the aforementioned two distributions are specified as follows:

- the Exponential Power Distribution [61]
  $$f\left(r\right)=\frac{\kappa }{2\sigma \Gamma (\frac{1}{\kappa })}\text{exp}\{-(\frac{|r-\mu }{\sigma }{)}^{\kappa }\}$$ (3)
  for −∞ < *r* < ∞, −∞ < *μ* < ∞, *σ* > 0 and *κ* > 0, where Γ(.) defines the gamma function, defined as $\Gamma \left(a\right)=\int_{0}^{\infty }t^{a-1}\text{exp}\left(-t\right)dt$.
- the Laplace Distribution [62]
  $$\begin{array}{c} f\left(r\right)=\frac{1}{2\sigma }\text{exp}(-\frac{\left|r-\mu \right|}{\sigma }) \end{array}$$ (4)
  for −∞ < *r* < ∞, −∞ < *μ* < ∞ and *σ* > 0.

The distributions were fitted by the method of Maximum Likelihood, using Mathematica to estimate the relevant parameters. Since estimating the standard errors for the Laplace and Subbotion distribution using Fisher information is not trivial (the Laplace distribution is unimodal (single-peaked) and is thus not continuously differentiable), standard errors of the parameters for the Laplace distribution were estimated using a bootstrap method. The bootstrap estimates the standard errors of parameters by drawing data points out of the dataset at random, replicating the same length of the dataset; it also estimates parameters using the maximum likelihood method. This procedure is repeated 10,000 times. The standard deviation is then mapped on the transposed list of bootstrap estimates. The same method was used to solve for standard errors for the Subbotin distribution. The results for all estimates are shown in S3 Table, with standard errors in parentheses. The results for the virtual intra-day currency exchange rate returns are displayed in S8 Table.

It is interesting to briefly interpret the special case for *κ* = 1 of the Subbotin distribution, where the Subbotion distribution equals a Laplace distribution. Hence, both distributions are nested [63]. As can be seen in S7 Fig, the first three virtual exchange rate shape parameters are slightly below unity, when considering the top of the double standard error bands shown in gray. However, USD/XRP and the shape parameter for intra-virtual currencies differ to those of first three somehow. The red dots describe the estimated parameter *κ*; the gray dots indicate positive and negative double standard errors. The results are remarkable for foreign exchange rates. The shape parameter is basically unity. As such, the shape may be best described by the Laplace distribution for traditional currencies, as well as for the most important virtual currencies. Intriguing are also the shape parameter values shown in S8 Table for the intra-day virtual currency data; except for USD/BTC, the *κ* values tend to be around unity.

To validate the visual impressions of S7 Fig, supporting goodness of fit tests were also conducted. These tests were the Kolmogorov-Smirnov (KS) and Anderson-Darling (AD) statistics, which are well-known and widely used methods for discriminating between fitted distributions. The results of the test statistics are given in S4 Table. In general, the smaller the value of the test statistics of the distribution, the better the distribution fits the data. The tests support the Laplace distribution best, considering the constraints of the Subbotin distribution (number of parameters / degrees of freedom), which must also be considered. The Laplace distribution is significant especially for almost all virtual and traditional currencies (daily log returns), and also achieves lower statistics values than the Subbotin distribution in some cases. The test statistics for the virtual currency intra-day data can be found in S9 Table.

More importantly, a likelihood ratio test was used to distinguish nested distributions, which is true for the Laplace and Subbotin distribution [63]. It can be introduced as follows: Let *L*_1_ be the maximum value of the likelihood without an additional assumption, and let *L*_0_ be the maximum value of the likelihood where the parameters are restricted and reduced in number, based on an assumption. In our case, we want to distinguish whether the Subbotin or Laplace distribution yield significantly different results. *L*_1_ displays the likelihood of the Laplace distribution and *L*_0_ the likelihood of the Subbotin distribution, where the shape parameter *κ* is fixed to unity. Then the ratio forms as λ = *L*_0_/*L*_1_ and *χ*^2^ can be calculated by *χ*^2^ = −2*ln*λ. If the calculated value is significantly higher than the counter value to the 100(1 − *α*) percentile point of a Chi-Square distribution with *k* degrees of freedom, we can reject the hypothesis that, in our case, the Subbotin distribution would yield significantly better results than the Laplace distribution. The Subbotin has three parameters and the Laplace two parameters, thus the degrees of freedom *k* = 1. As can be seen in S5 Table, the Laplace distribution yields better, but more parsimonious, test results than the Subbotin distribution. Therefore, the likelihood ratio test fails to confirm that the Subbotin distribution yields significantly better results than the nested Laplace distribution, when exploring virtual and intra-virtual currencies with daily returns. Thus the likelihood ratio test also backs the previously conducted goodness of fit tests. This robustness will be heightened when visually inspecting the outcome in the Results. The ratio test results for the intra-day virtual currencies are displayed and briefly discussed in S10 Table.

Finally, to examine the comparability of daily virtual and intra-virtual to traditional foreign currencies, the two stylized facts shortly introduced in in Public perception and rising academic interest in recent years are reviewed. An examination is therefore undertaken to determine whether exchange rate increments are uncorrelated, and whether changing volatility regimes results in long-range dependence. In statistics, the autocorrelation, or serial correlation, of a random process is the Pearson correlation between values of the process at different times, as a function of the two times or the time lag. This function is applied versatilely in financial and economic time series analysis [64, 65]. If these phenomena can be confirmed, it would be another indication that virtual, intra-virtual and actual foreign exchange rates share some statistical and distributional properties.

## Results

**Fig 1:**
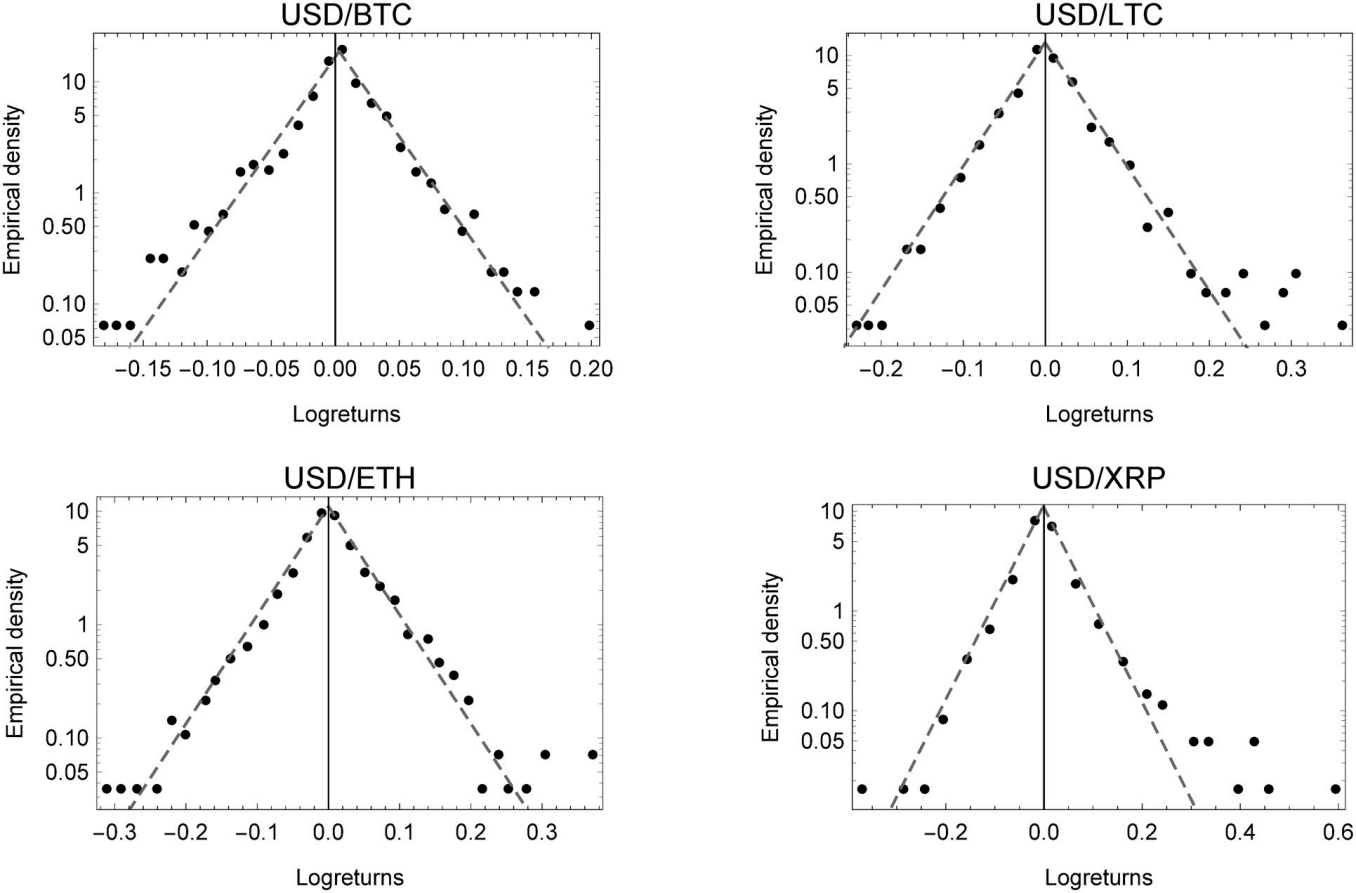
Empirical densities of virtual currencies. Empirical densities of currency exchange rate log-returns for the virtual currencies.

Fig 1 shows the binned empirical densities of the virtual exchange rate log-returns, displaying the characteristic tent-shape of a Laplace distribution on a semi-log scale. Dispersions of the log-return in the lower log-scale are more frequent and extreme, similar to the intra-virtual group, but less so than the traditional currency group described below. As such, USD/BTC provides a more consistent picture, whereas the log-returns of USD/LTC and USD/XRP, for example, exhibit extreme events on the right-hand side of the distribution. The results obtained for the intra-day virtual exchange rate returns are shown in S11 Fig.

**Fig 2:**
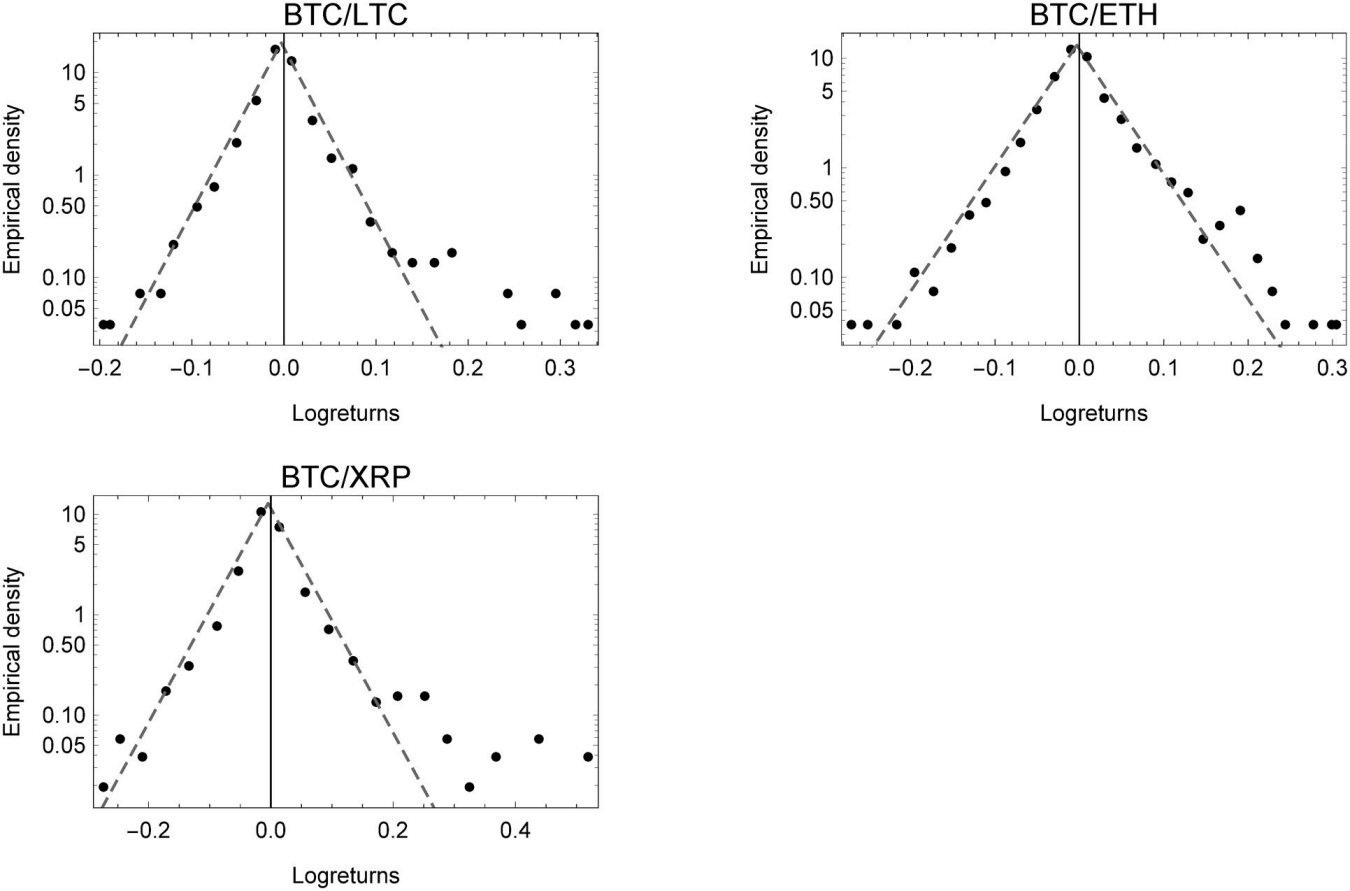
Empirical densities of intra-virtual currencies. Empirical densities of currency exchange rate log-returns for the intra-virtual currencies.

Fig 2 shows the binned empirical densities of the intra-virtual exchange rate log-returns, also displaying the characteristic tent-shape of a Laplace distribution on a semi-log scale. As with virtual log-returns, intra-virtual log-returns are sharper (more leptokurtic) than the Laplace itself, when considering the shape parameter of the Subbotin distribution. Moreover, the values of parameter *κ* are further from unity than virtual currencies. However, intra-virtual daily log-returns seem to fit a superimposed Laplace distribution quite well. Comparing the log-return on the x-axis to that of virtual and foreign currencies, we see that they are more similar to the virtual currencies, but more extreme than foreign currencies, while still inheriting the same functional form.

**Fig 3:**
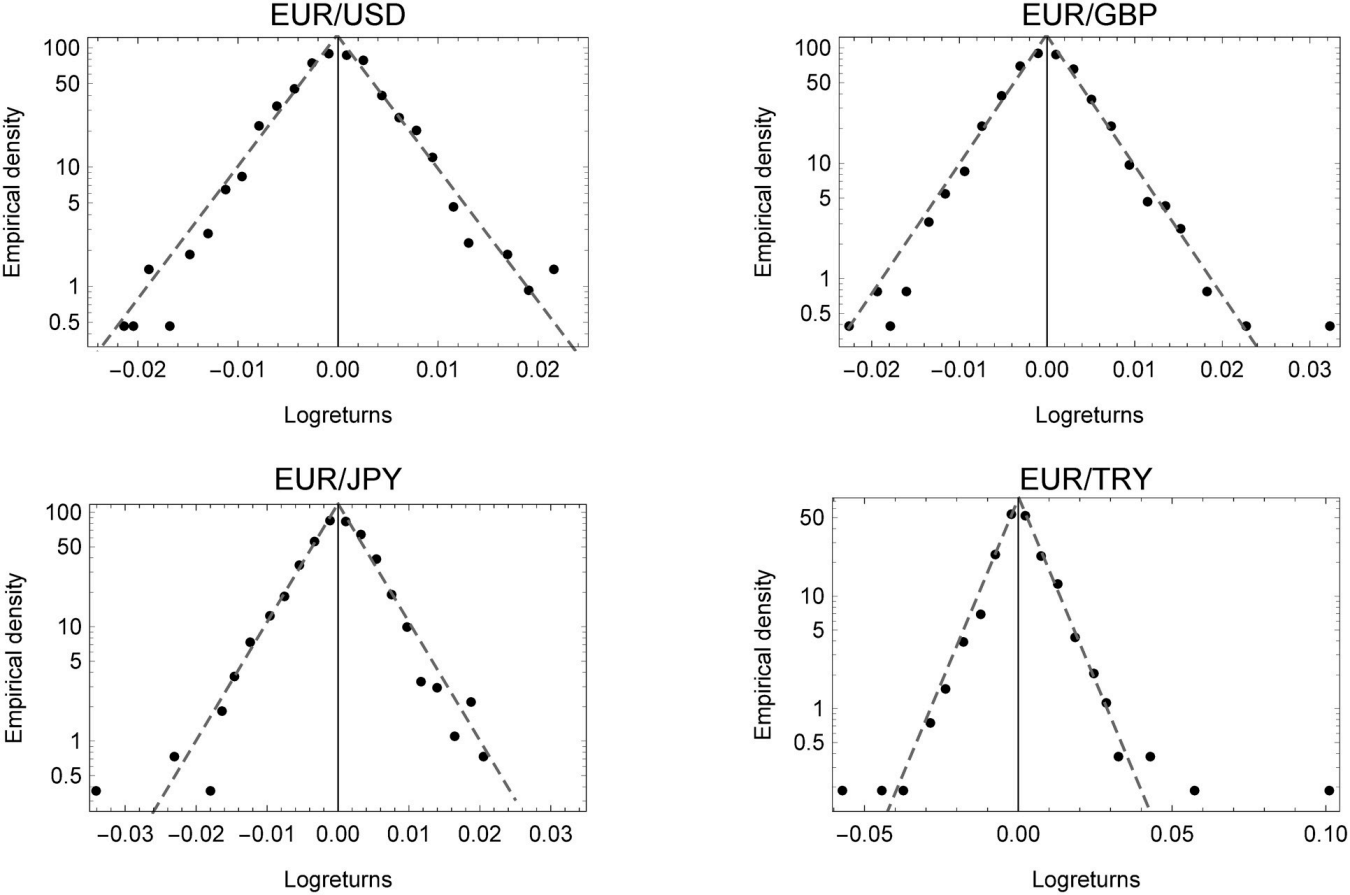
Empirical densities of foreign currencies. Empirical densities of foreign currency exchange rate log-returns for the currencies valued against the Euro.

The binned empirical densities of foreign exchange rates on a semi-log scale are shown in Fig 3. Strikingly, as already described, the shape parameter *κ* perfectly matches the Laplace nested in the Subbotin distribution. It therefore, comes as no surprise that the results support the findings of [52] from the late 1980s for foreign exchange rate returns. Moreover, comparing the three plots, the analysis shows that daily log-returns of all currency types, share tent-shaped empirical densities, in spite of their differing mode of formation. This peculiar property also applies, for example, to firm growth or profit rates, as explained above. Also, when comparing the daily empirical densities of virtual currencies in Fig 1 to those of foreign currencies in Fig 3, we can see that extreme values of log-returns occur much more often and in a more extreme fashion, and therefore generate heavier tails. Moreover, even the hype of 2017 and 2018 leads to significant differences (as can be seen in the lower part of the semi-log plots), yet the same functional form prevails.

Before pooling the different groups, they were standardized by subtracting the mean and dividing by the standard deviation to avoid multi-modality in the shown pooled empirical densities. After pooling the different exchange rate log-returns contained in one group, it is vital to note that the general tent-shape and their distribution regularities prevail, while the shape parameter *κ* remains almost constant for virtual and intra-virtual currencies, slightly increasing for foreign currencies; this still points to a Laplace distribution.

**Fig 4:**
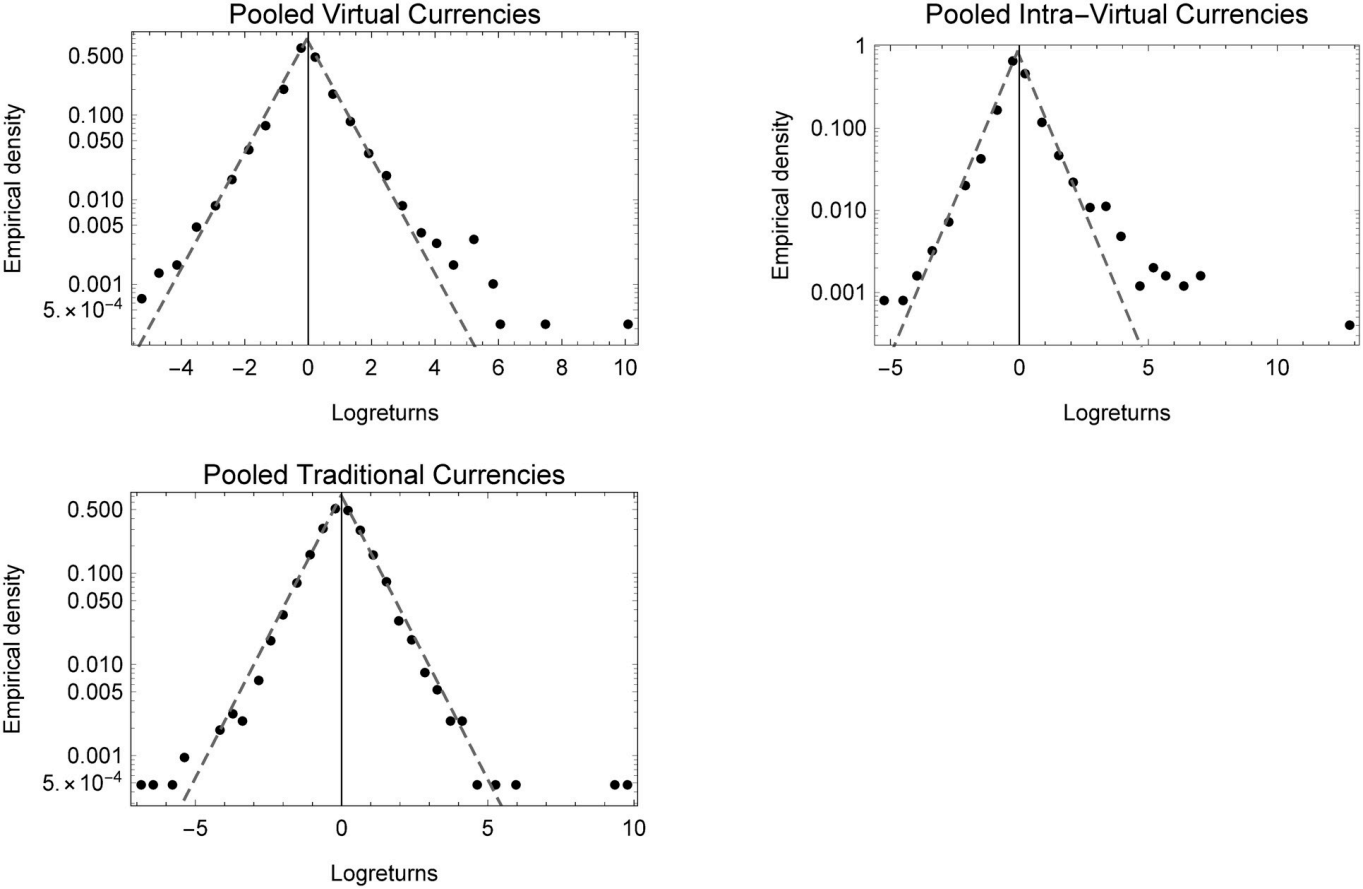
Empirical densities of standardized and pooled currencies. Empirical densities of standardized and pooled currency log-returns for all three groups of virtual, intra-virtual and foreign currency exchange rates.

Fig 4 shows the results obtained. Both virtual, and intra-virtual currencies have many more outliers in the lower scale of the empirical density plot, especially to the right indicating the high returns during the hype. Moreover, this may be a clear indication of the volatility and upheavals faced by these currencies in recent years.

But what is implied by the fact that all currency pairs of log-returns point, some more strongly than others, to a Laplace distribution? One explanation could be that traders of virtual currencies follow positive feedback strategies (buy when prices rise, sell when prices fall). This means that, “if rational speculators early buying triggers positive feedback trading, then an increase in the number of forward-looking speculators can increase volatility about fundamentals [66].” This explanation also holds for firm growth rates, for example, as explained in [8]. This may be a suitable explanation, as many agents followed the trend of rising virtual currency prices, especially over the last two years, when the hype around virtual currencies gathered pace. It can also result from extrapolative expectations about the future price of an asset or trend shaping, driving prices up exponentially. A similar phenomenon was shown by [67] for Bitcoin, where two positive feedback loops were identified, implying a constant increase in price. However, these explanations fail to explain the sudden negative shocks that drove down Bitcoin prices. Now, that Bitcoin and other virtual currencies have lost a substantial share of their value over a few months, the aspect of sudden negative shocks may also be investigated in the future.

But why are log returns rather tent-shaped? The Laplace distribution is also called double exponential distribution, because it can be viewed as two exponential distributions brought together, back-to-back. This makes the Laplace distribution more leptokurtic than normal distribution and results in a single towering peak. Following [43], the Laplace random variable can be represented as the difference between two i.i.d. exponential random variables. According to [68], this is important because “the difference, and hence the Laplace distribution, provides a characterization of the error in a timing device that is under periodic excitation”.

To compare the properties of exchange rate returns of traditional, foreign currencies with virtual and intra-virtual currencies, further stylized facts of logarithmic returns were analyzed, as explained in Public perception and rising academic interest in recent years. The plots on the left side of S8, S9 and S10 Figs show the autocorrelation function of virtual, intra-virtual and foreign exchange rate returns. Analogously the results for the intra-day virtual currencies can be found in S12 Fig. As in a wide range of different speculative markets, the universal property of uncorrelated raw returns can also be found in virtual and intra-virtual currencies. This means that the evolution of all three types of exchange rate resemble a random walk. Interpreting this factor economically, one could also argue that, in the sense of [53], the (weak) condition of the efficient market hypothesis (EMH) is fulfilled, as arbitrage does not seem possible, and new information is immediately factored in the price-forming process.

Interestingly, this is not the case for the USD/LTC and USD/XRP exchange rate returns, all intra-day virtual some daily intra-virtual currencies and the EUR/TRY, at least on visual inspection. Also, the Ljung-Box and Box-Pierce tests reject the null hypothesis that the data are uncorrelated to lag-eight at the 5 percent level. The same holds for all intra-virtual exchange rate returns. The results of the Box-Ljung and Box-Pierce tests are given in S6 Table for the daily data and in S11 Table for the virtual intra-day data. Hence, the autocorrelation encountered in all five daily currency pairs and the intra-day data would open the door for statistical arbitrage, a simple strategy with positive expected earnings [53]. With the high transaction costs mentioned in Public perception and rising academic interest in recent years, this arbitrage strategy may not be a suitable explanation for this effect. Nonetheless, the unequal possession of several individuals in this exclusive market may be an explanation. As is well known, in several markets a disproportionately small number of individuals accumulate large sums of virtual currencies [69]. Hence, if their market capital becomes overpowering, they may be able to capitalize on their position by transferring outstanding amounts of capital, while still leveraging the transaction investment.

Another stylized fact that helps to distinguish whether virtual and intra-virtual exchange rates resemble actual foreign currencies is long-range dependence or long memory. This means that periods of low volatility are followed by periods of high volatility; they can be recognized by the fact that the autocorrelation function of absolute returns is positive, and decays slowly. Here, it is true for virtual and intra-virtual currencies, as can be seen on the right side of S8, S9 and S10 Figs and also for intra-day virtual currencies of S12 Fig. Intriguingly, the long-range dependence of virtual and intra-virtual currencies decays much more quickly, and the correlation is far higher than in traditional currencies, rather resembling other, more speculative asset categories. There may be two reasons for this: First of all, the virtual currency market is far more speculative, and is driven by trend-following agents who rely on increasing prices, while avoiding the market or not knowing about it. Examples are the volatility increase in USD/LTC or BTC/LTC trade in early 2017, as can be seen in and. Another explanation may be that, in contrast to the foreign exchange market, the virtual currency market is more exclusive and thus contains fewer participants by several orders of magnitude, in contrast to the large number of agents in the foreign exchange market.

## Discussion and conclusion

The virtual and intra-virtual currency market share the rather turbulent dynamics of a traditional foreign exchange market, and even more extremely to some extent. It has been empirically shown that virtual, intra-virtual and foreign currency log-returns are tent-shaped, have the characteristics of a Laplace distribution at the semi-log scale, and share the same functional form. However, this non-Gaussianity questions conditions of the central limit theorem, more specifically the independence assumption. Furthermore, virtual and intra-virtual currencies exhibit greater volatility, fatter tails and steeper towering peaks than regular foreign currencies, while following the stylized facts of asset returns in a more extreme fashion to some extent. It may therefore be better to view virtual and intra-virtual currencies as a speculative investment, as noted by [18]. The results also contribute to a recent strand of literature comparing and classifying virtual currencies to other asset categories. Since autocorrelation has also been identified in daily log-returns for one virtual currency and all intra-virtual currencies, statistical arbitrage may be possible. This supports the findings of [38], identifying arbitrage strategies due to co-movement.

To answer the question of what is left after the hype, it can be said that daily log-returns of all currency types share, tent-shaped empirical densities, one of the characteristics of a Laplace distribution at the semi-log scale, in spite of the differing mode of formation, technology and speculative intensity in recent years. This peculiar property is shared with several asset types and applies to firm growth and profit rates, for example [8–10]. Moreover, the results highlight the fact that virtual and traditional currencies hold the same functional form, even after the hype of 2018.

Although a direct comparison of the results is not possible due to the difference in frequencies it is important to note that when shifting the frequency to intra-day price observations, the distributional properties of virtual currencies seem to change, too. Even though the Laplace and Subbotin distribution seem to fit the data well in the tent-shaped middle, they fail to describe the numerous extreme events happening within a day being pictured by the tails. Therefore, finding a distribution which fits higher frequency data of virtual and traditional data as good as the Laplace does for daily log-returns would be a valuable investigation in the future. Yet, due to the lag of free available intra-day data of traditional currencies, this might be a cumbersome task. Furthermore, as evidence for autocorrelation has been presented, it would be interesting to investigate whether statistical arbitrage strategies can be or have been realized for virtual currencies, or whether this is merely an artifact in the perception or representation of any information used and induced by the technology involved. It would also be interesting to explore whether there are any interdependencies between the currency groups. To this end, it would be interesting to see how, and if, idiosyncratic external shocks (Brexit, the presidential election in the US) influenced the price of foreign and virtual currencies, for instance. A first guess would be that there is no such effect for virtual and intra-virtual currencies, while there is for foreign currencies.

Summing up, what is left after the hype is a peculiar statistical regularity shared with many asset classes. But, in the future, virtual currencies may be of interest as an asset, decoupled from the conventional financial system. However the risk, and therefore uncertainty, in the virtual exchange rate market seems to be higher due to its speculative nature, and must be valued with caution, especially following the extreme hype in 2017 and the massive crash of 2018.

## Supporting information

S1 Fig Price time series of virtual currencies. Closing price development of virtual currency exchange rates.
(PDF)

S2 Fig Price time series of intra-virtual currencies. Closing price development of intra-virtual currency exchange rates.
(PDF)

S3 Fig Price time series of foreign currencies. Closing price development of foreign currency exchange rates.
(PDF)

S4 Fig Volatility clustering in virtual currency log-returns. Volatility clustering in log-returns of the virtual to real currency exchange rate group.
(PDF)

S5 Fig Volatility clustering in intra-virtual currency log-returns. Volatility clustering in log-returns of the intra-virtual to virtual currency exchange rate group.
(PDF)

S6 Fig Volatility clustering in foreign currency log-returns. Volatility clustering in log-returns of foreign currency exchange rate group.
(PDF)

S7 Fig Shape parameter *κ* of Subbotin distribution. Shape parameter *κ* of Subbotin distribution for virtual, intra-virtual and foreign currency exchange rate log-returns (daily).
(PDF)

S8 Fig Autocorrelation functions of virtual currencies. Autocorrelation function of raw and absolute returns of virtual currency exchange rates.
(PDF)

S9 Fig Autocorrelation functions of intra-virtual currencies. Autocorrelation function of raw and absolute returns of intra-virtual currency exchange rates.
(PDF)

S10 Fig Autocorrelation functions of foreign currencies. Autocorrelation function of raw and absolute returns of foreign currency exchange rates.
(PDF)

S11 Fig Empirical densities of intra-day virtual currencies. Empirical densities of currency exchange rate log-returns for the intra-day virtual currencies.
(PDF)

S12 Fig Autocorrelation functions of intra-day virtual currencies. Autocorrelation function of raw and absolute returns of intra-day virtual currency exchange rates.
(PDF)

S1 Table Market capitalization and trade volume information for selected virtual currencies—January 2018. (PDF)

S2 Table Market capitalization and trade volume information for selected virtual currencies—January 2017. (PDF)

S3 Table Fitted distributions, parameter estimates and standard errors. (PDF)

S4 Table Kolmogorow-Smirnow and Anderson-Darling goodness of fit test statistics for fitted distributions. (PDF)

S5 Table Likelihood ratio test results. (PDF)

S6 Table Ljung-Box and Box-Pierce test results. (PDF)

S7 Table Descriptive statistics of intra-day virtual exchange rates. (PDF)

S8 Table Fitted distributions, parameter estimates and standard errors for intra-day exchange rates. (PDF)

S9 Table Kolmogorow-Smirnow and Anderson-Darling goodness of fit test statistics for fitted distributions on virtual currency intra-day data. (PDF)

S10 Table Likelihood ratio test results for virtual intra-day data. (PDF)

S11 Table Ljung-Box and Box-Pierce test results for virtual intra-day data. (PDF)
