# Supplementary figures and images for: What’s left after the hype? An empirical approach comparing the distributional properties of traditional and virtual currency exchange rates
Source: PLoS One. 2019 Jul 26;14(7):e0220070. doi: 10.1371/journal.pone.0220070 (PMC6660129; doi:10.1371/journal.pone.0220070)

USD/BTC

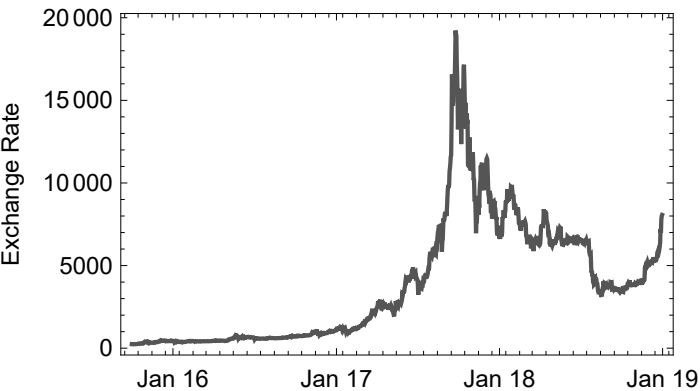

USD/LTC

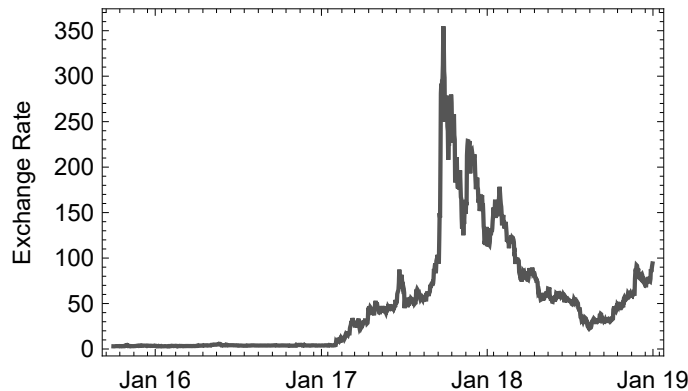

USD/ETH

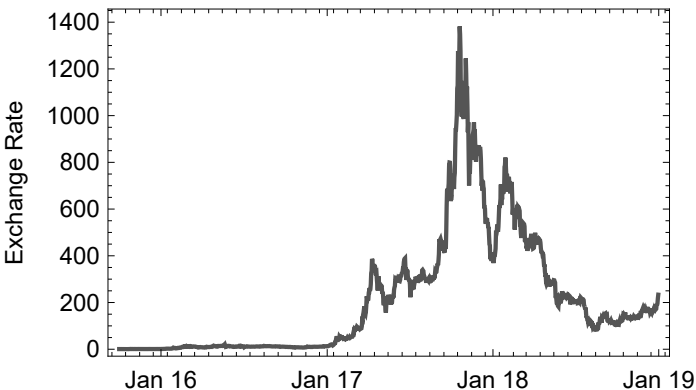

USD/XRP

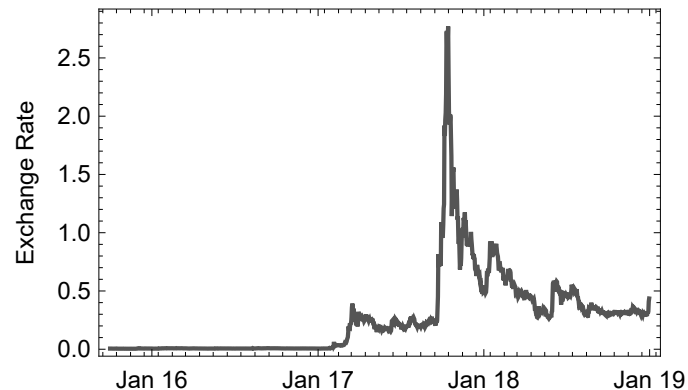

Supplement: S1 Fig — Closing price development of virtual currency exchange rates. (PDF) [file pone.0220070.s001.pdf]

BTC/LTC

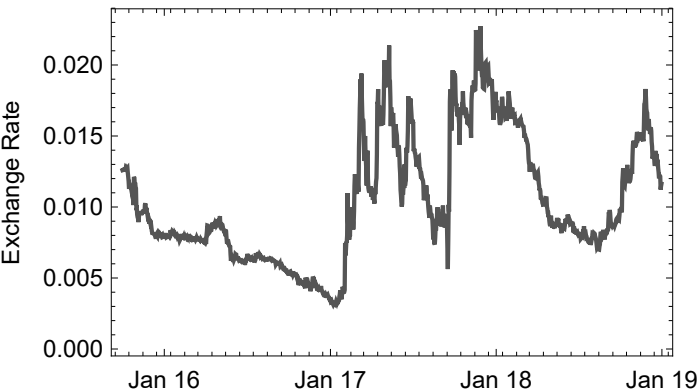

BTC/ETH

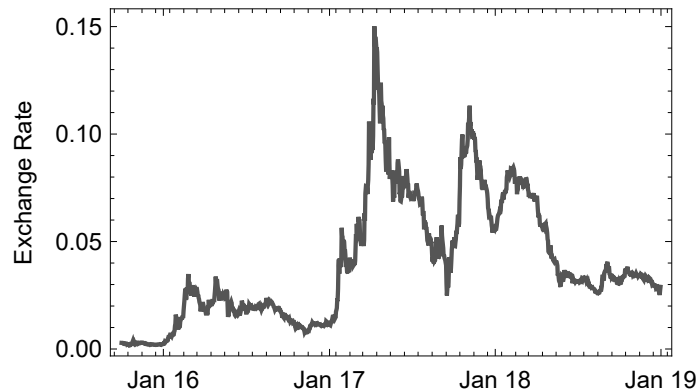

BTC/XRP

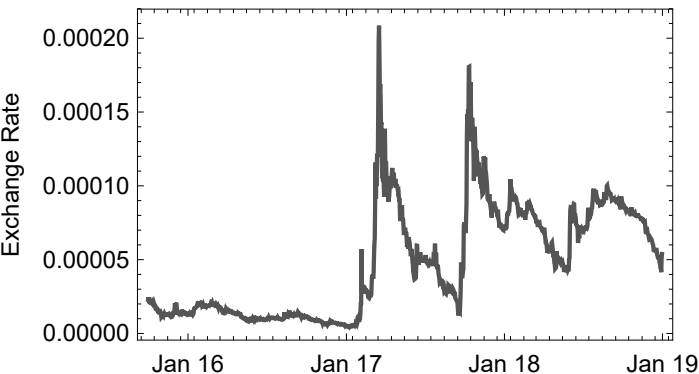

Supplement: S2 Fig — Closing price development of intra-virtual currency exchange rates. (PDF) [file pone.0220070.s002.pdf]

EUR/USD

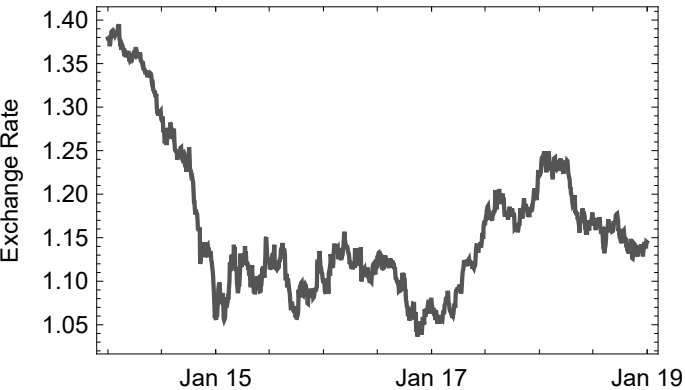

EUR/GBP

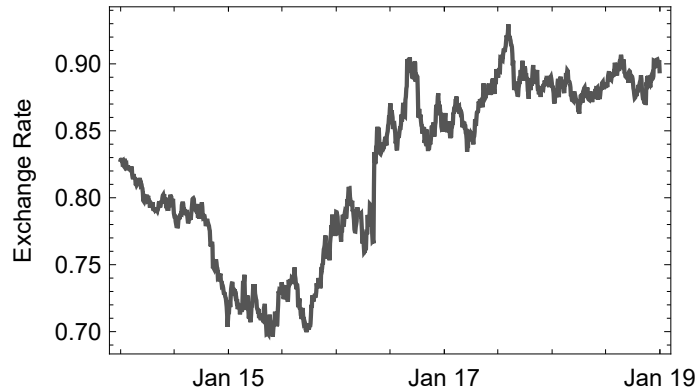

EUR/JPY

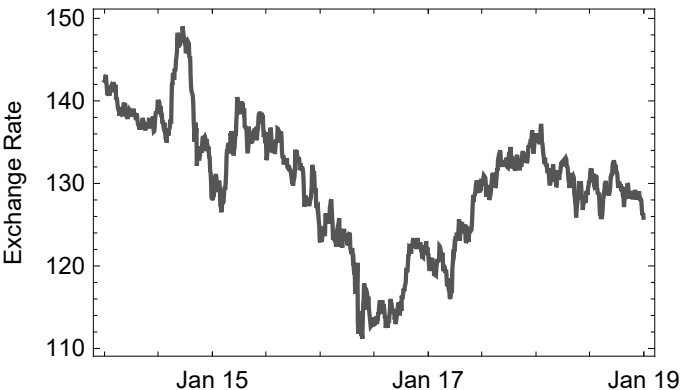

EUR/TRY

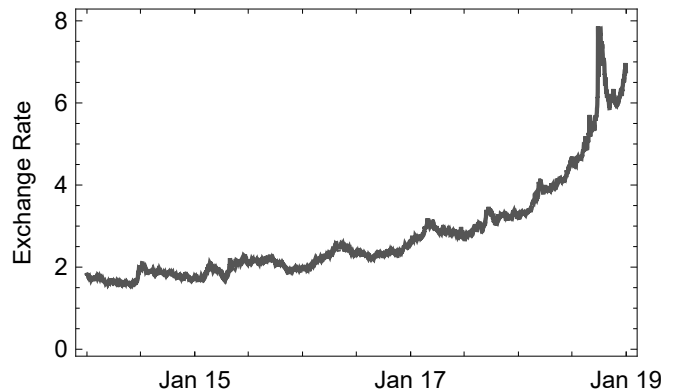

Supplement: S3 Fig — Closing price development of foreign currency exchange rates. (PDF) [file pone.0220070.s003.pdf]

USD/BTC

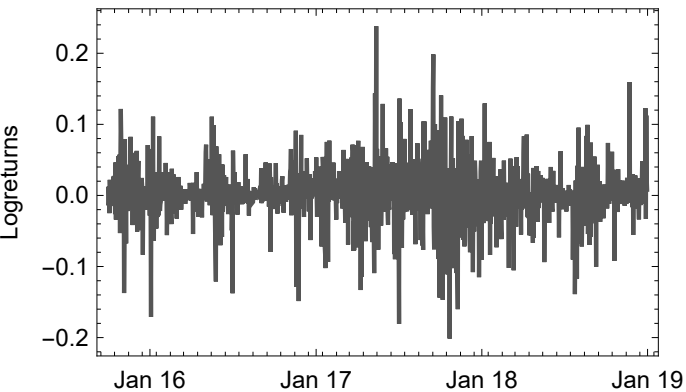

USD/LTC

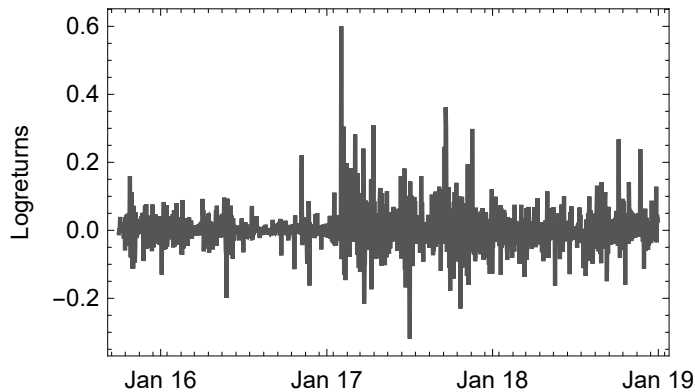

USD/ETH

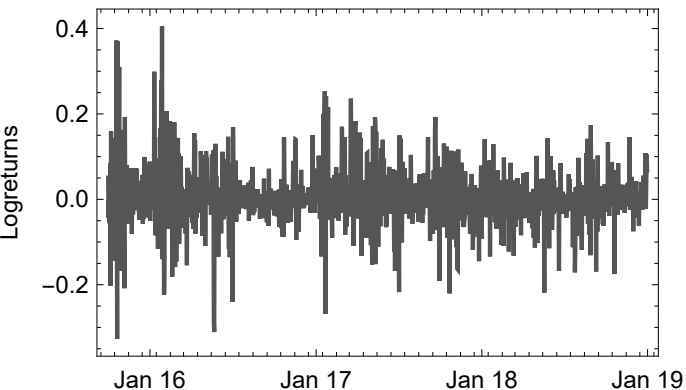

USD/XRP

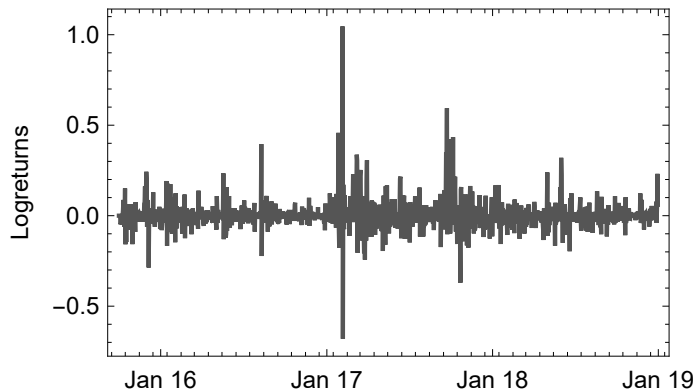

Supplement: S4 Fig — Volatility clustering in log-returns of the virtual to real currency exchange rate group. (PDF) [file pone.0220070.s004.pdf]

BTC/LTC

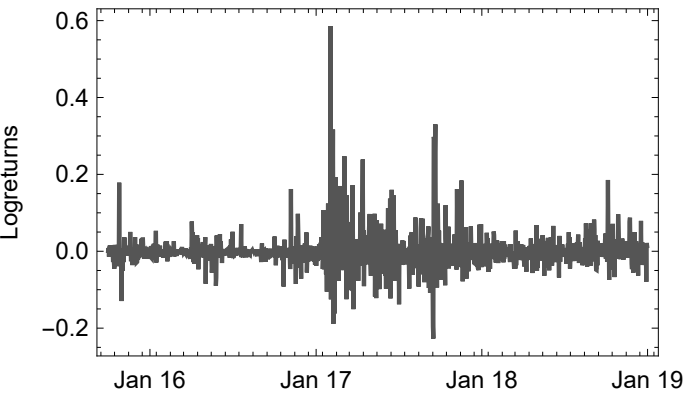

BTC/ETH

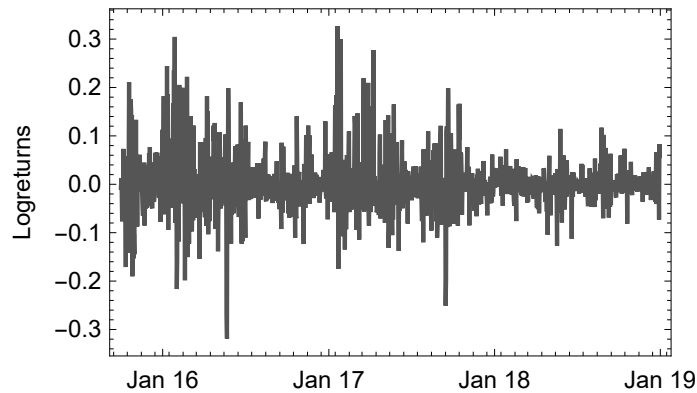

BTC/XRP

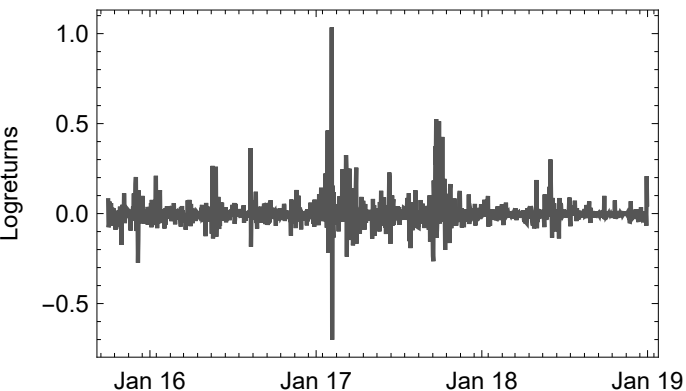

Supplement: S5 Fig — Volatility clustering in log-returns of the intra-virtual to virtual currency exchange rate group. (PDF) [file pone.0220070.s005.pdf]

EUR/USD

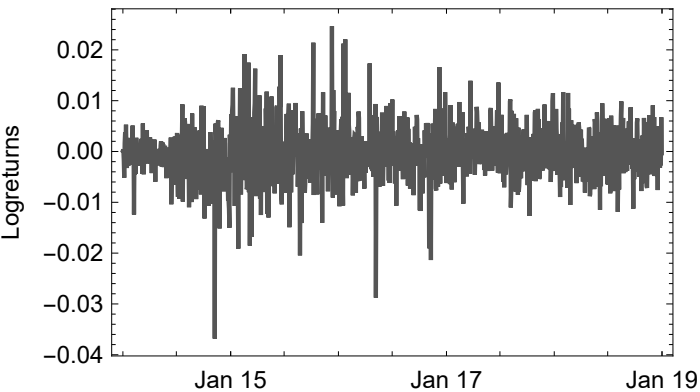

EUR/GBP

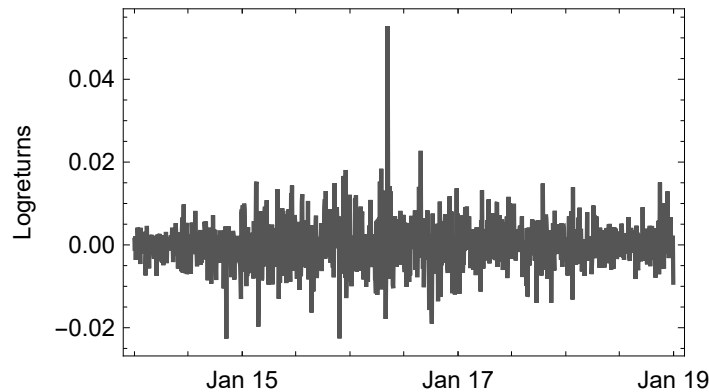

EUR/JPY

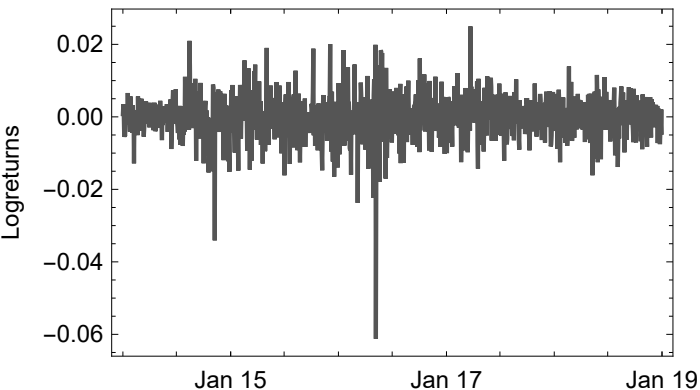

EUR/JPY

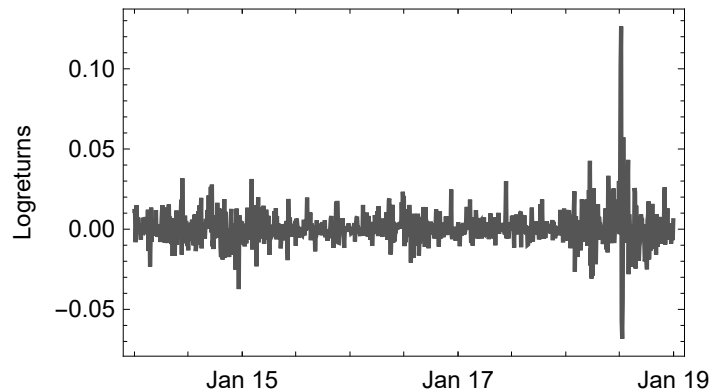

Supplement: S6 Fig — Volatility clustering in log-returns of foreign currency exchange rate group. (PDF) [file pone.0220070.s006.pdf]

Shape parameter  $\kappa$  of Subbotin DistributionShape parameter  $\kappa$ 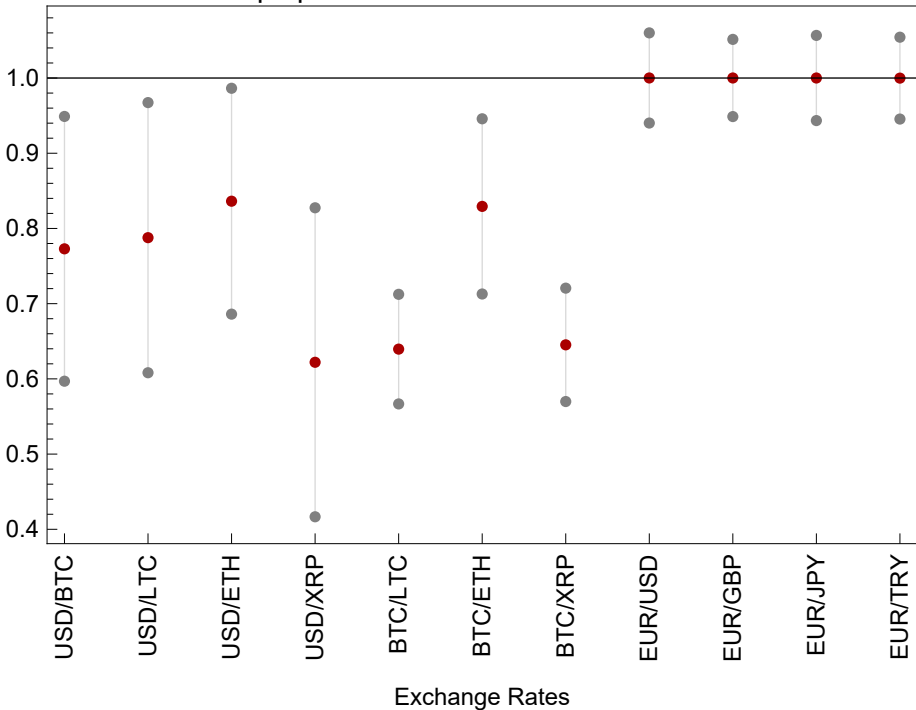

Supplement: S7 Fig — Shape parameter κ of Subbotin distribution for virtual, intra-virtual and foreign currency exchange rate log-returns (daily). (PDF) [file pone.0220070.s007.pdf]

USD/BTC

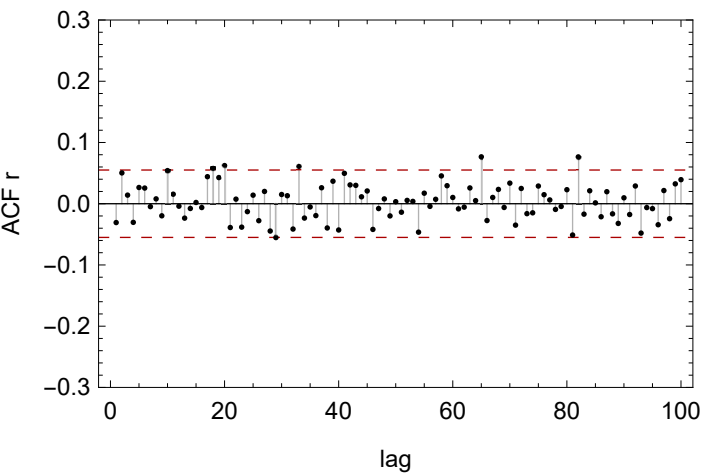

USD/BTC

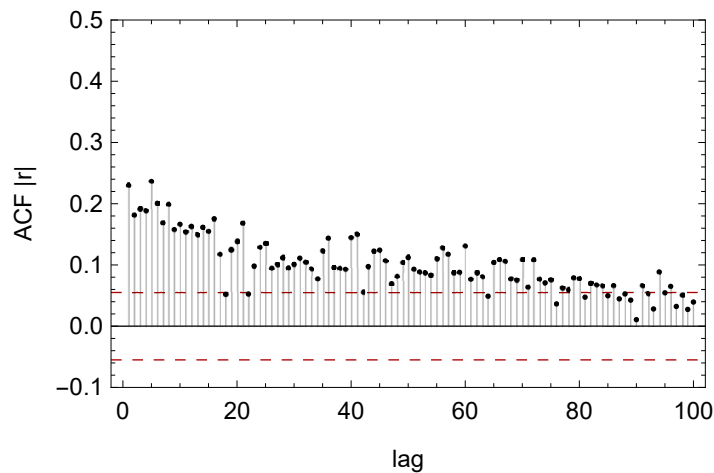

USD/LTC

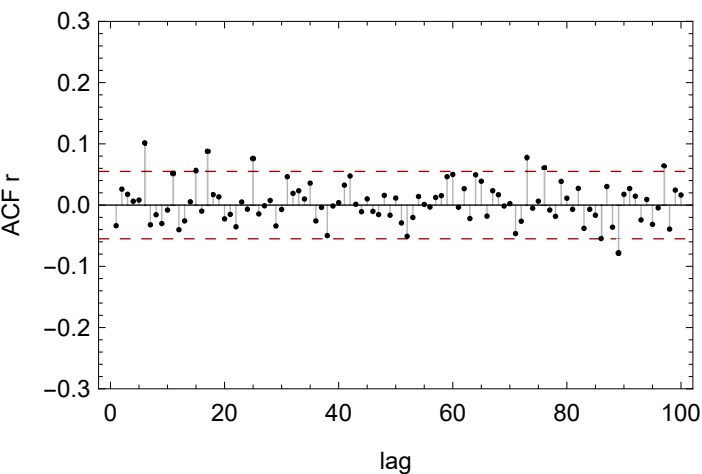

USD/LTC

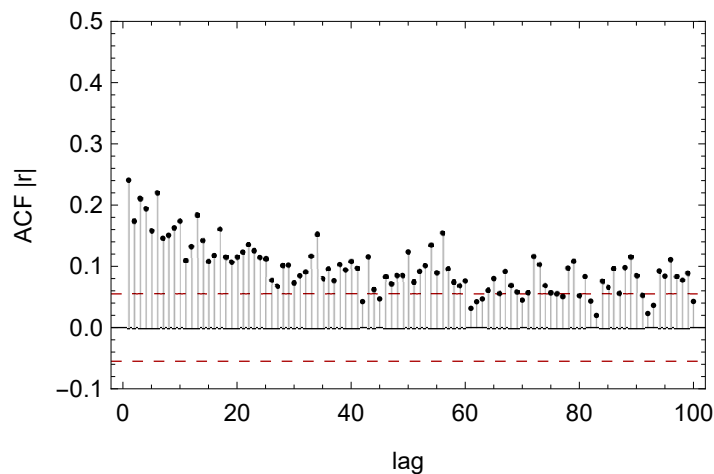

USD/ETH

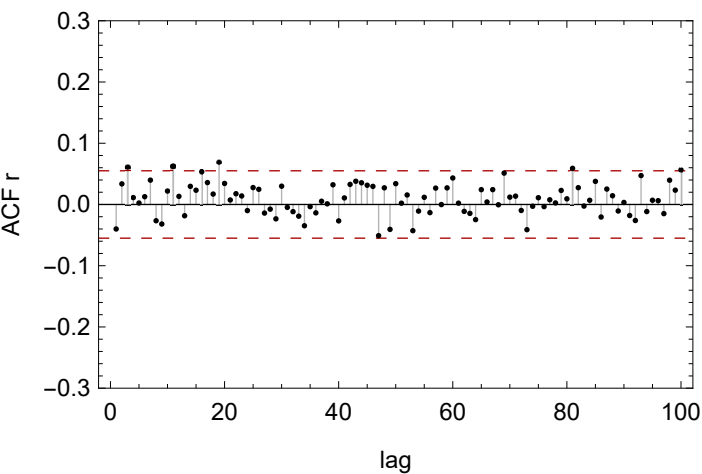

USD/ETH

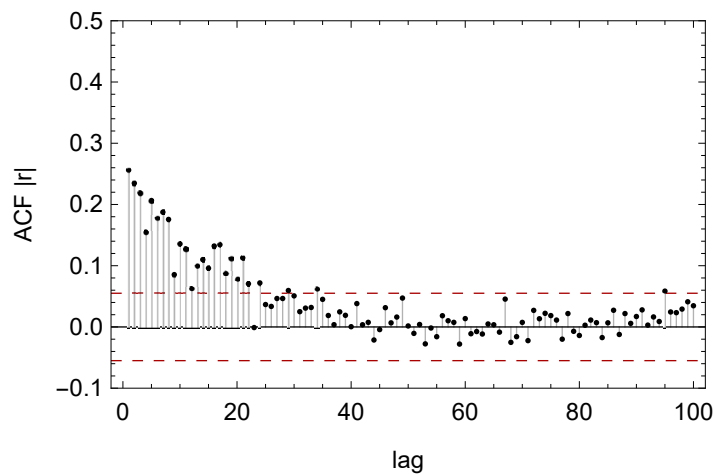

USD/XRP

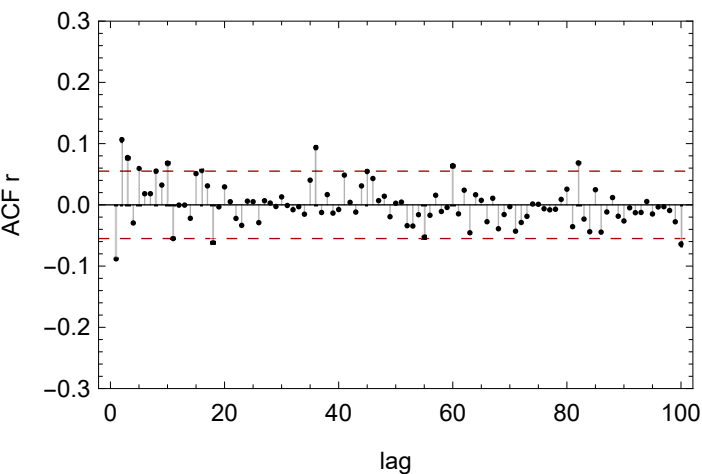

USD/XRP

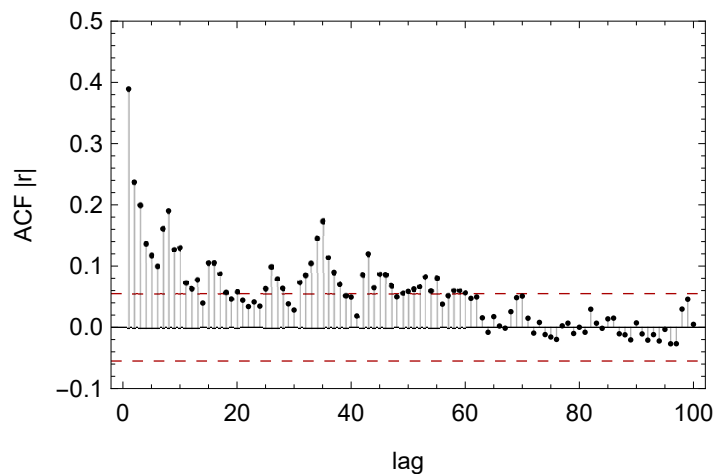

Supplement: S8 Fig — Autocorrelation function of raw and absolute returns of virtual currency exchange rates. (PDF) [file pone.0220070.s008.pdf]

BTC/LTC

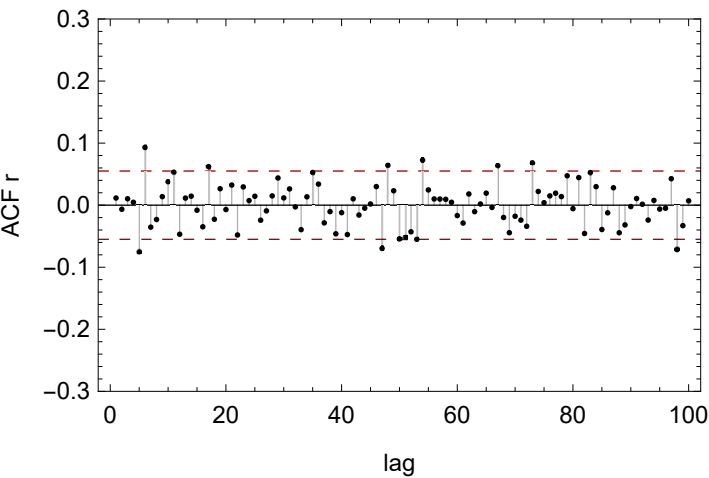

BTC/LTC

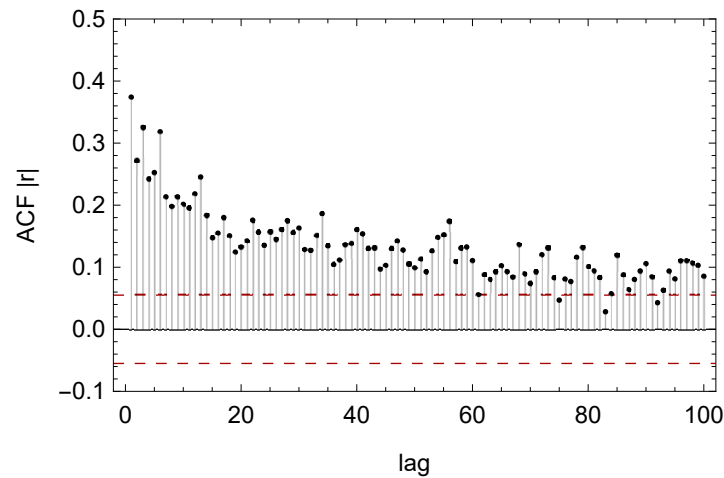

BTC/ETH

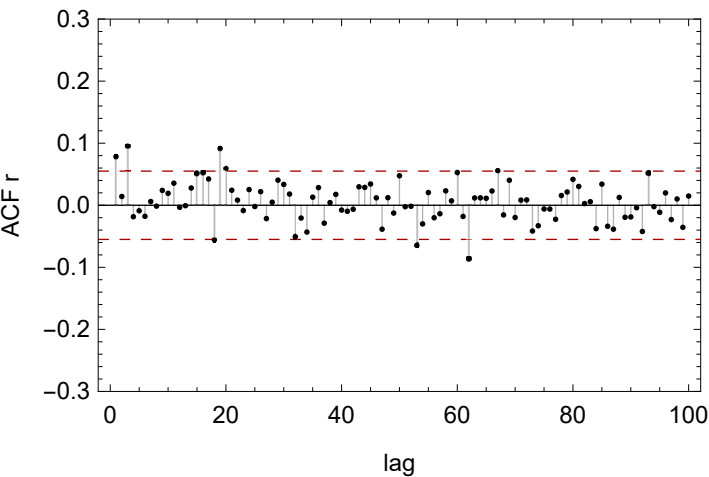

BTC/ETH

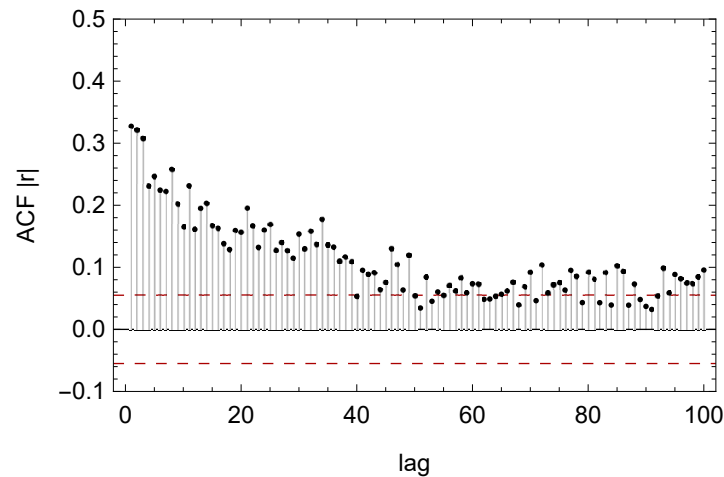

BTC/XRP

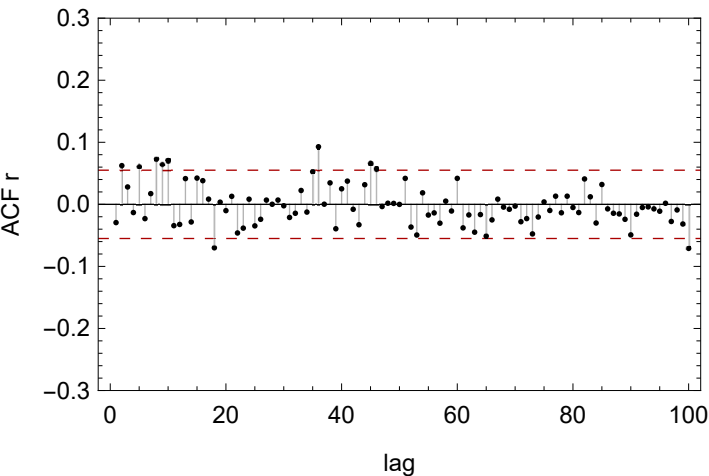

BTC/XRP

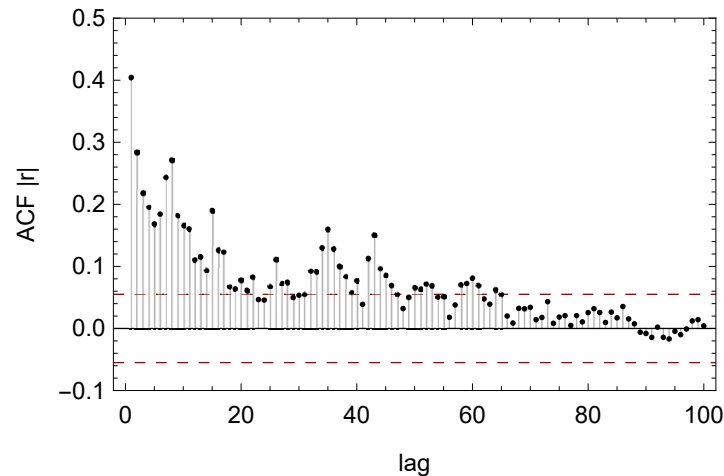

Supplement: S9 Fig — Autocorrelation function of raw and absolute returns of intra-virtual currency exchange rates. (PDF) [file pone.0220070.s009.pdf]

EUR/USD

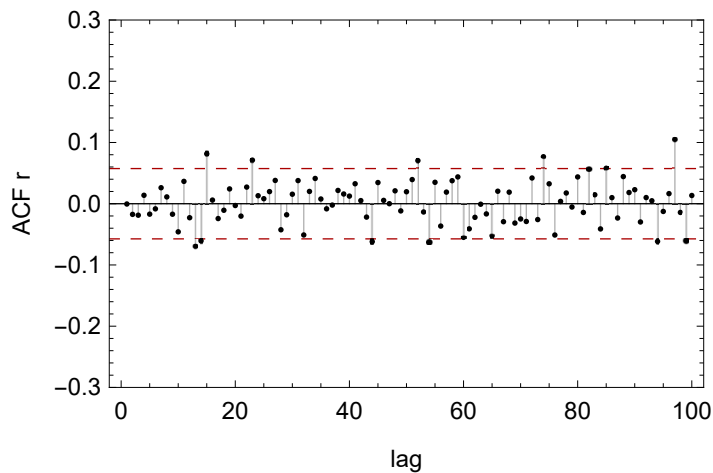

EUR/USD

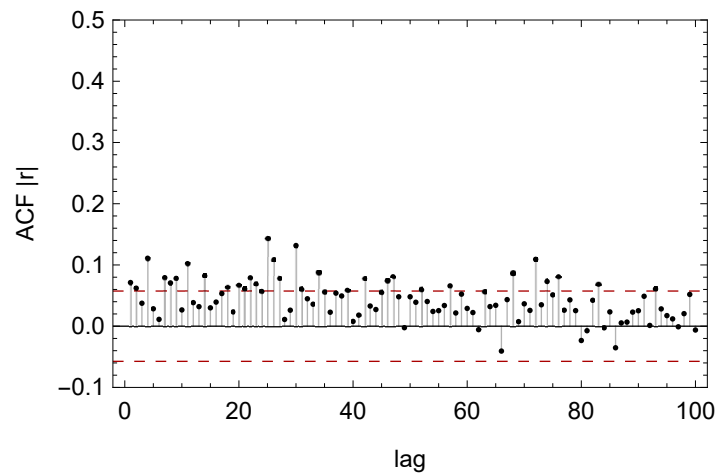

EUR/GBP

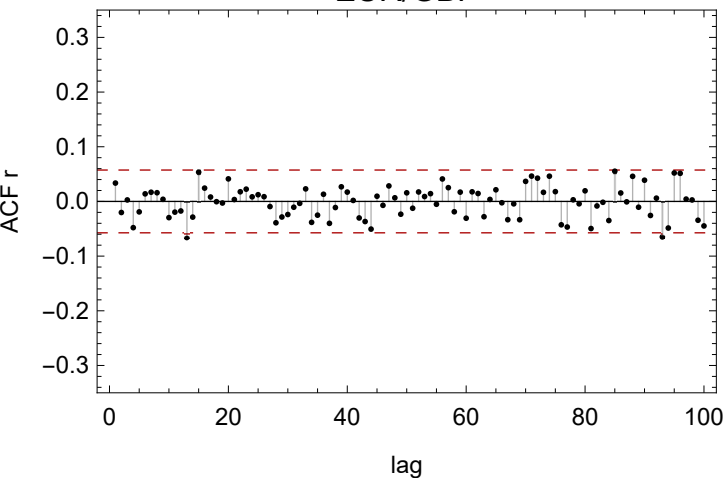

EUR/GBP

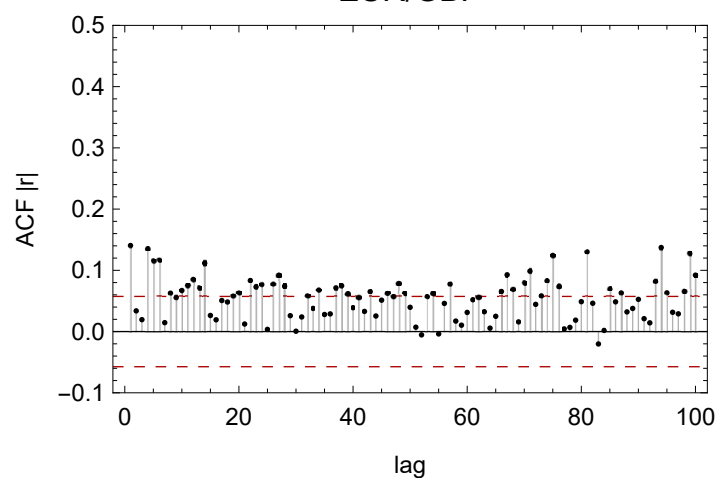

EUR/JPY

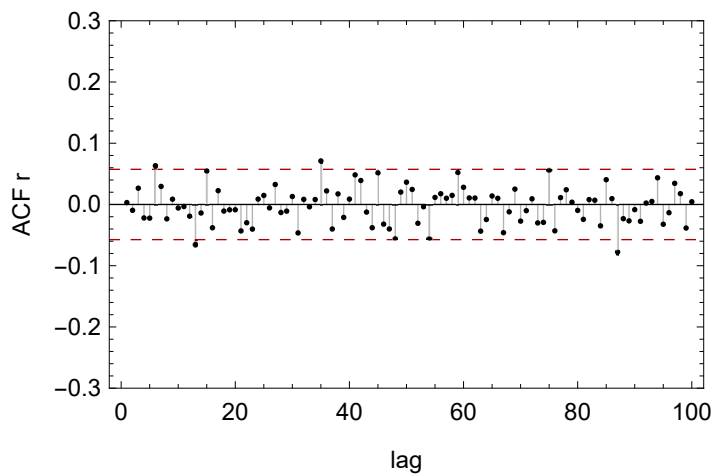

EUR/JPY

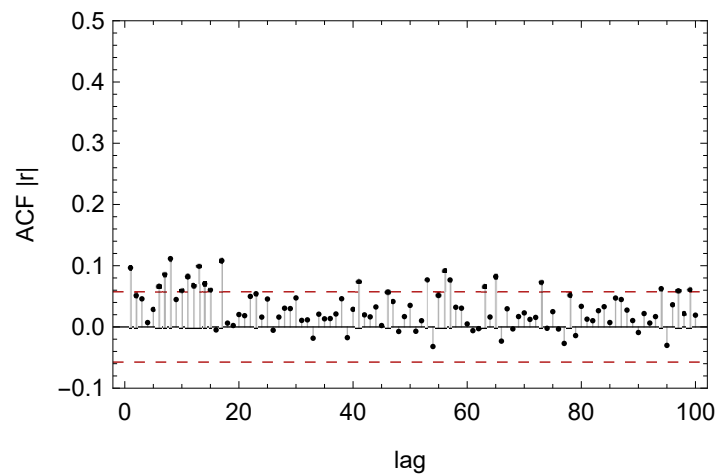

EUR/TRY

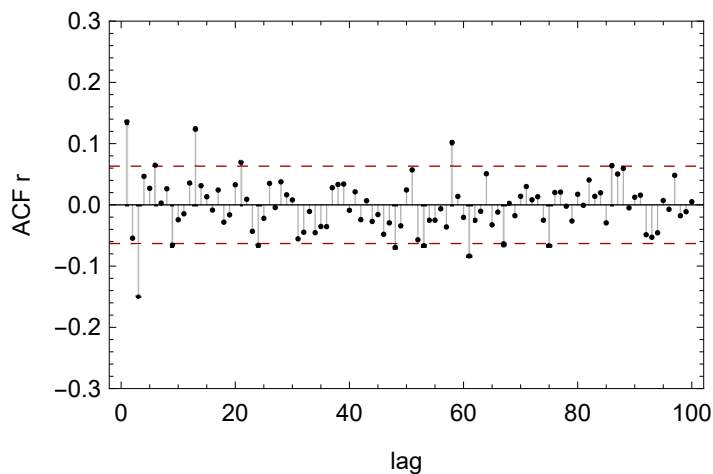

EUR/TRY

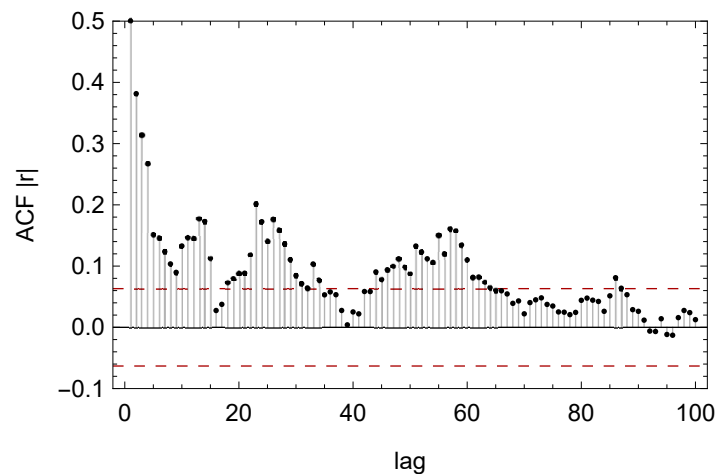

Supplement: S10 Fig — Autocorrelation function of raw and absolute returns of foreign currency exchange rates. (PDF) [file pone.0220070.s010.pdf]

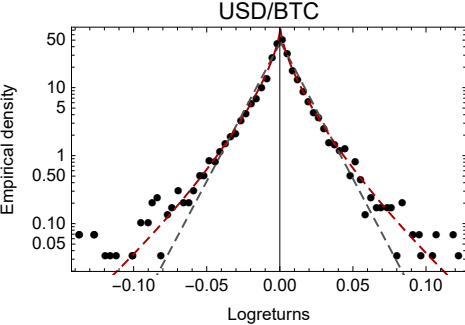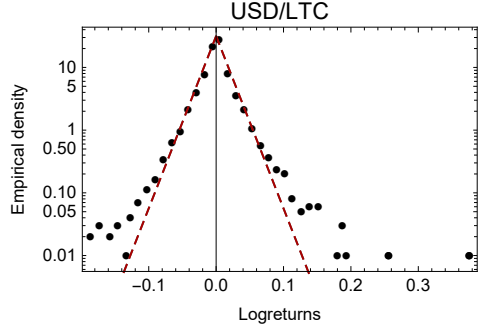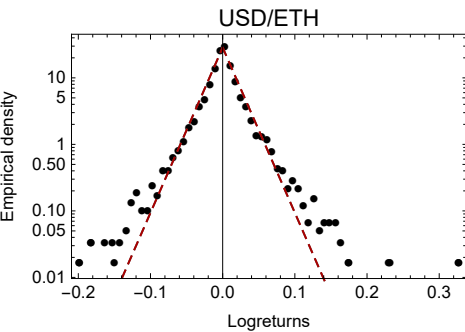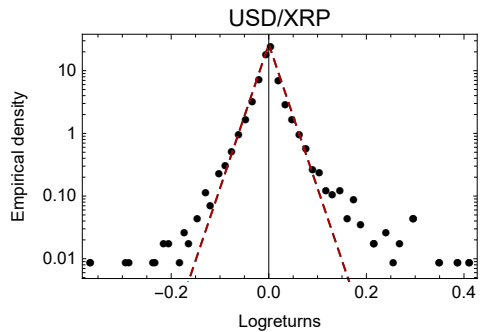

-- Laplace Distribution    - - Subbotin Distribution

Supplement: S11 Fig — Empirical densities of currency exchange rate log-returns for the intra-day virtual currencies. (PDF) [file pone.0220070.s011.pdf]

USD/BTC

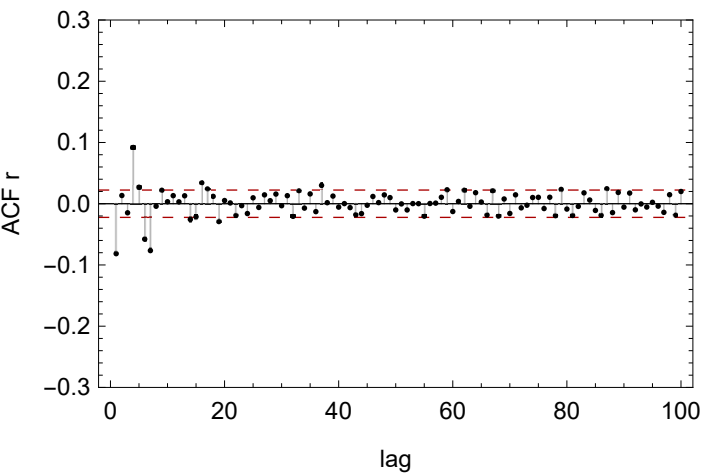

USD/BTC

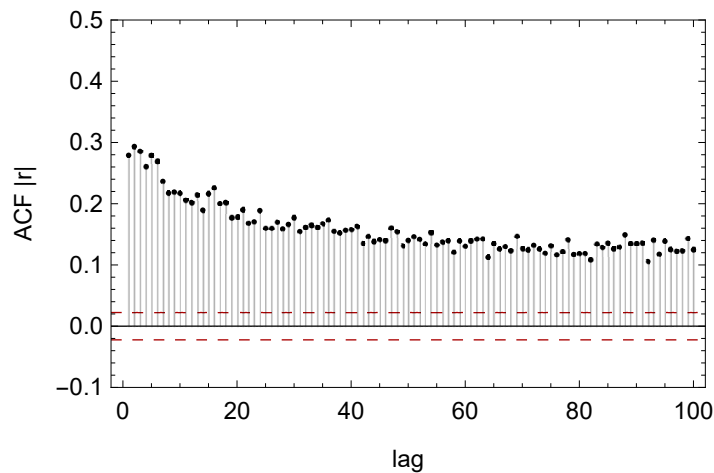

USD/LTC

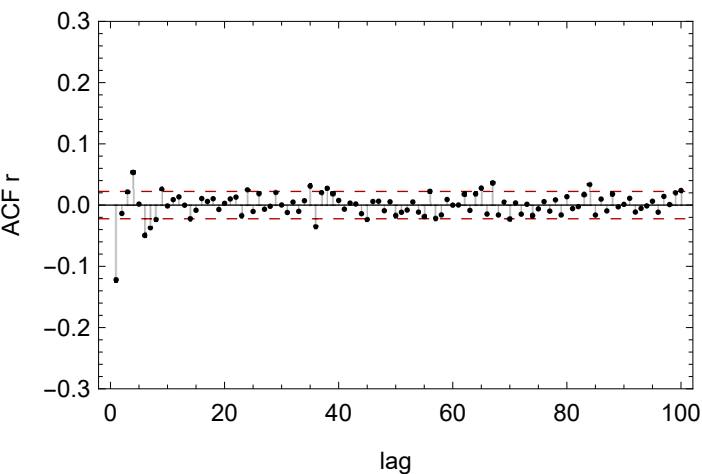

USD/LTC

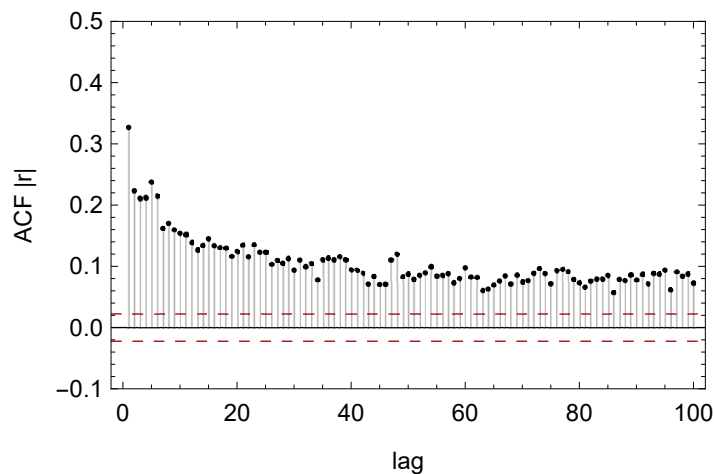

USD/ETH

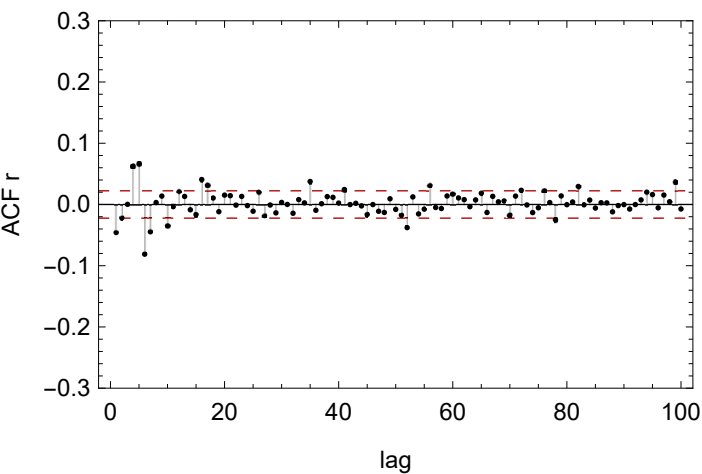

USD/ETH

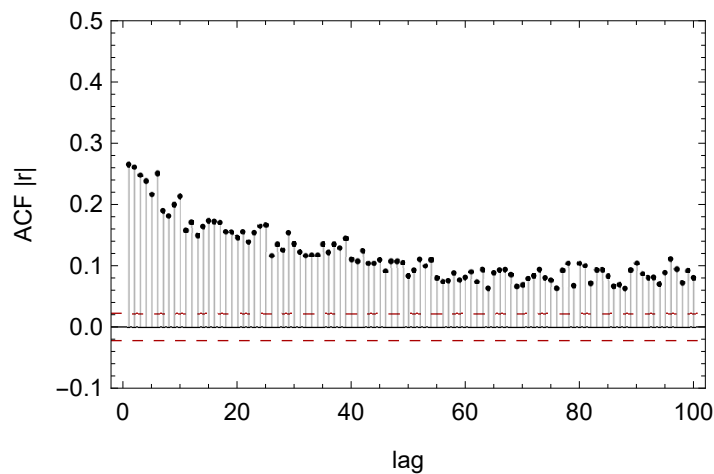

USD/XRP

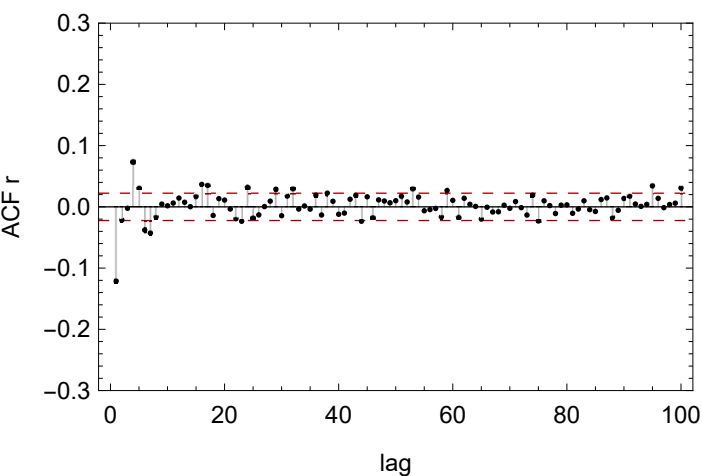

USD/XRP

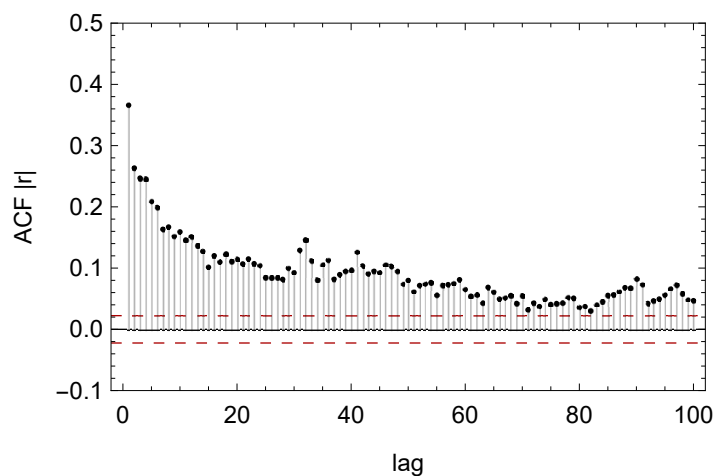

Supplement: S12 Fig — Autocorrelation function of raw and absolute returns of intra-day virtual currency exchange rates. (PDF) [file pone.0220070.s012.pdf]
