# Supplementary material for: What’s left after the hype? An empirical approach comparing the distributional properties of traditional and virtual currency exchange rates
Source: PLoS One. 2019 Jul 26;14(7):e0220070. doi: 10.1371/journal.pone.0220070 (PMC6660129; doi:10.1371/journal.pone.0220070)
Supplement: S1 Table — (PDF) [file pone.0220070.s013.pdf]

S1 Table.

Currency	Market Cap	Volume (24h)	Available Supply	Maximum Supply
Bitcoin	$227,578,237,659	$11,534,500,000	16,802,262 BTC	21,000,000 BTC
Ethereum	$127,953,096,946	$5,216,660,000	96,973,835 ETH	—
Ripple	$70,987,542,244	$1,846,320,000	38,739,142,811 XRP	100,000,000,000 XRP
Litecoin	$13,051,269,334	$1,041,820,000	54,751,458 LTC	84,000,000 LTC

Market capitalization and trade volume information for selected virtual currencies - January 2018.
