# Supplementary material for: What’s left after the hype? An empirical approach comparing the distributional properties of traditional and virtual currency exchange rates
Source: PLoS One. 2019 Jul 26;14(7):e0220070. doi: 10.1371/journal.pone.0220070 (PMC6660129; doi:10.1371/journal.pone.0220070)
Supplement: S2 Table — (PDF) [file pone.0220070.s014.pdf]

S2 Table.

Currency	Market Cap	Volume (24h)	Available Supply	Maximum Supply
Bitcoin	$15,078,898,496	$95,527,900	16,135,862 BTC	21,000,000 BTC
Ethereum	$936,697,745	$8,675,030	88,424,437 ETH	—
Ripple	$235,014,889	$409,345	36,856,524,148 XRP	100,000,000,000 XRP
Litecoin	$200,552,099	$18,074,500	49,582,331 LTC	84,000,000 LTC

Market capitalization and trade volume information for selected virtual currencies - January 2017.
