# Supplementary material for: What’s left after the hype? An empirical approach comparing the distributional properties of traditional and virtual currency exchange rates
Source: PLoS One. 2019 Jul 26;14(7):e0220070. doi: 10.1371/journal.pone.0220070 (PMC6660129; doi:10.1371/journal.pone.0220070)
Supplement: S3 Table — (PDF) [file pone.0220070.s015.pdf]

S3 Table.

	Laplace Distribution		Subbotin Distribution		
	$\hat{\mu}$ (SE)	$\hat{\sigma}$ (SE)	$\hat{\kappa}$ (SE)	$\hat{\mu}$ (SE)	$\hat{\sigma}$ (SE)
USD/BTC	0.00316 (0.00059)	0.02747 (0.00088)	0.77291 (0.08801)	0.00309 (0.00057)	0.02433 (0.00147)
USD/LTC	0.00061 (0.00089)	0.03822 (0.00132)	0.787747 (0.08980)	$-1.14 \cdot 10^{-9}$ (0.00105)	0.03411 (0.00192)
USD/ETH	0.00003 (0.00112)	0.04709 (0.00149)	0.83623 (0.07508)	$-7.73 \cdot 10^{-9}$ (0.00105)	0.04339 (0.00218)
USD/XRP	-0.00134 (0.00122)	0.04649 (0.00192)	0.62206 (0.10271)	$-1.03 \cdot 10^{-9}$ (0.00143)	0.036448 (0.00304)
BTC/LTC	-0.00321 (0.00063)	0.02557 (0.00111)	0.63956 (0.03643)	-0.00316 (0.00089)	0.02005 (0.00074)
BTC/ETH	-0.00287 (0.00130)	0.04009 (0.00130)	0.82933 (0.05824)	-0.00315 (0.00149)	0.03676 (0.00150)
BTC/XRP	-0.00390 (0.00117)	0.04106 (0.00189)	0.64525 (0.03769)	-0.00345 (0.00176)	0.03232 (0.00119)
EUR/USD	-0.00009 (0.00016)	0.00390 (0.00010)	1.00009 (0.02997)	-0.00009 (0.00015)	0.0.00390 (0.00010)
EUR/GBP	-0.00007 (0.00017)	0.00385 (0.00010)	1.00007 (0.02566)	-0.00007 (0.00017)	0.00385 (0.00011)
EUR/JPY	0. (0.00017)	0.00419 (0.00011)	1.00004 (0.02831)	0.00014 (0.00016)	0.00419 (0.00011)
EUR/TRY	0.00010 (0.00018)	0.00629 (0.00022)	0.99999 (0.02721)	0.00016 (0.00021)	0.00664 (0.00027)
Pooled Virtual Currencies	-0.04111 (0.00583)	0.63885 (0.01109)	0.75841 (0.01801)	-0.04111 (0.00266)	0.55943 (0.01580)
Pooled Intra-Virtual Currencies	-0.06758 (0.00965)	0.58214 (0.01335)	0.68894 (0.01615)	-0.06589 (0.01677)	0.48008 (0.00877)
Pooled Foreign Currencies	-0.01220 (0.01383)	0.69571 (0.01027)	1.07443 (0.03900)	0.01174 (0.01268)	0.71637 (0.01210)

Fitted distributions, parameter estimates and standard errors. Table notes: Variables are log-returns of the respective currencies. The Pooled Exchange Rates are standardized for mean zero and standard deviation one.
