# Supplementary material for: What’s left after the hype? An empirical approach comparing the distributional properties of traditional and virtual currency exchange rates
Source: PLoS One. 2019 Jul 26;14(7):e0220070. doi: 10.1371/journal.pone.0220070 (PMC6660129; doi:10.1371/journal.pone.0220070)
Supplement: S4 Table — (PDF) [file pone.0220070.s016.pdf]

S4 Table.

	Kolmogorow-Smirnow		Anderson-Darling	
	Laplace	Subbotin	Laplace	Subbotin
USD/BTC	0.04139	0.02105*	2.33460*	0.58989*
USD/LTC	0.03362*	0.09708	1.44721*	19.2695
USD/ETH	0.03601*	0.02703*	2.42895*	1.65442*
USD/XRP	0.04145	0.04801	5.00501	2.82935
BTC/LTC	0.05466	0.02262*	7.62011	1.44359*
BTC/ETH	0.03585*	0.03016*	3.61771	.583180
BTC/XRP	0.05478	0.04081	8.52987	2.97168
EUR/USD	0.03188*	0.03188*	1.59082*	1.59082*
EUR/GBP	0.02998*	0.02998*	1.45344*	1.45344*
EUR/JPY	0.03294*	0.03294*	1.65519*	1.65519*
EUR/TRY	0.02894*	0.03668*	1.47207*	2.62343

Kolmogorow-Smirnow and Anderson-Darling goodness of fit test statistics for fitted distributions.
*: Significant for a significance level of $\alpha = 0.05$.
