# Supplementary material for: What’s left after the hype? An empirical approach comparing the distributional properties of traditional and virtual currency exchange rates
Source: PLoS One. 2019 Jul 26;14(7):e0220070. doi: 10.1371/journal.pone.0220070 (PMC6660129; doi:10.1371/journal.pone.0220070)
Supplement: S5 Table — (PDF) [file pone.0220070.s017.pdf]

S5 Table.

**Currency**	$-2\ln\lambda$**-Value**	χ^2**-Benchmark**	**Statement**
USD/BTC	18.553	3.841	Reject
USD/LTC	-730.876	3.841	Accept
USD/ETH	8.311	3.841	Reject
USD/XRP	79.182	3.841	Reject
BTC/LTC	77.626	3.841	Reject
BTC/ETH	9.392	3.841	Reject
BTC/XRP	75.266	3.841	Reject
EUR/USD	$1.09 \cdot 10^-12$	3.841	Accept
EUR/GBP	1.085	3.841	Accept
EUR/JPY	$0.11 \cdot 10^-12$	3.841	Accept
EUR/TRY	3.595	3.841	Accept

Likelihood ratio test results. Table notes: Degree of freedom $k = 1$. Since the Subbotion distribution equals a Laplace distribution for $\kappa = 1$, it comes as no surprise that the Likelihood ratio test yields better results because the κ parameters reported in S3 Table are close to unity.
