# Supplementary material for: What’s left after the hype? An empirical approach comparing the distributional properties of traditional and virtual currency exchange rates
Source: PLoS One. 2019 Jul 26;14(7):e0220070. doi: 10.1371/journal.pone.0220070 (PMC6660129; doi:10.1371/journal.pone.0220070)
Supplement: S6 Table — (PDF) [file pone.0220070.s018.pdf]

S6 Table.

	**Ljung-Box**		**Box-Pierce**	
	Statistic	P-Value	Statistic	P-Value
USD/BTC	7.07728	0.52831	7.04688	0.53158
USD/LTC	21.5188	0.00588*	21.3853	0.00619*
USD/ETH	11.5488	0.17249	11.4915	0.17537
USD/XRP	41.1377	$1.96 \cdot 10^{-}6$*	40.9697	$2.11 \cdot 10^{-}6$*
BTC/LTC	21.3024	0.00638*	21.1649	0.00672*
BTC/ETH	20.3187	0.00919*	20.2461	0.00944*
BTC/XRP	19.4751	0.01251*	19.3628	0.01303*
EUR/USD	2.45668	0.96372	2.44254	0.96435
EUR/GBP	6.03101	0.64375	6.00286	0.64691
EUR/JPY	8.82376	0.35738	8.76778	0.36226
EUR/TRY	56.2427	$2.52 \cdot 10^{-}9$*	56.4588	$2.29 \cdot 10^{-}9$*

Ljung-Box and Box-Pierce test results. Table notes: The null hypothesis that data is uncorrelated to lag 8 is or is not rejected at the 5 percent level. *: These tests are rejected at the 5 percent level.
