# Supplementary material for: What’s left after the hype? An empirical approach comparing the distributional properties of traditional and virtual currency exchange rates
Source: PLoS One. 2019 Jul 26;14(7):e0220070. doi: 10.1371/journal.pone.0220070 (PMC6660129; doi:10.1371/journal.pone.0220070)
Supplement: S7 Table — (PDF) [file pone.0220070.s019.pdf]

S7 Table.

Currency	N	Mean	SD	Median	Skewness	Kurtosis
USD/BTC	8021	0.00044	0.01748	0.00048	-0.19794	14.0661
USD/LTC	8021	0.00045	0.02765	0.	0.58395	69.3499
USD/ETH	8021	0.00073	0.02867	0.	0.49880	18.0121
USD/XRP	8021	0.00054	0.03520	0.	1.05859	57.2758

Descriptive statistics of intra-day virtual exchange rates.

Table notes: Variables are log-returns of the respective currencies.
