# Supplementary material for: What’s left after the hype? An empirical approach comparing the distributional properties of traditional and virtual currency exchange rates
Source: PLoS One. 2019 Jul 26;14(7):e0220070. doi: 10.1371/journal.pone.0220070 (PMC6660129; doi:10.1371/journal.pone.0220070)
Supplement: S8 Table — (PDF) [file pone.0220070.s020.pdf]

S8 Table.

	**Laplace Distribution**		**Subbotin Distribution**		
	$\hat{\mu}$ (SE)	$\hat{\sigma}$ (SE)	$\hat{\kappa}$ (SE)	$\hat{\mu}$ (SE)	$\hat{\sigma}$ (SE)
USD/BTC	0.00048 (0.00011)	0.01071 (0.00015)	0.67661 (0.01741)	0.00034 (0.00021)	0.00877 (0.00015)
USD/LTC	0. ($6.31 \cdot 10^{-}11$)	0.01579 (0.00025)	1. ($2.34 \cdot 10^{-}10$)	0. ($4.91 \cdot 10^{-}12$)	0.01579 (0.00025)
USD/ETH	0. (0.00003)	0.01747 (0.00025)	1. (0.14374)	0. ($1.57 \cdot 10^{-}9$)	0.01747 (0.00145)
USD/XRP	0. (0.)	0.01887 (0.00032)	1. (0.)	0. (0.)	0.01887 (0.00032)

Fitted distributions, parameter estimates and standard errors for intra-day exchange rates.
Table notes: Variables are log-returns of the respective currencies.
