# Supplementary material for: What’s left after the hype? An empirical approach comparing the distributional properties of traditional and virtual currency exchange rates
Source: PLoS One. 2019 Jul 26;14(7):e0220070. doi: 10.1371/journal.pone.0220070 (PMC6660129; doi:10.1371/journal.pone.0220070)
Supplement: S9 Table — (PDF) [file pone.0220070.s021.pdf]

S9 Table.

	**Kolmogorow-Smirnow**		**Anderson-Darling**	
	Laplace	Subbotin	Laplace	Subbotin
USD/BTC	0.07611	0.02196	66.1883	4.46575
USD/LTC	0.04523	0.04523	38.7224	38.7224
USD/ETH	0.04893	0.04893	29.3384	29.3384
USD/XRP	0.06409	0.06409	62.2389	62.2389

Kolmogorow-Smirnow and Anderson-Darling goodness of fit test statistics for fitted distributions on virtual currency intra-day data.
*: Significant for a significance level of $\alpha = 0.05$.
