# Supplementary material for: What’s left after the hype? An empirical approach comparing the distributional properties of traditional and virtual currency exchange rates
Source: PLoS One. 2019 Jul 26;14(7):e0220070. doi: 10.1371/journal.pone.0220070 (PMC6660129; doi:10.1371/journal.pone.0220070)
Supplement: S10 Table — (PDF) [file pone.0220070.s022.pdf]

S10 Table.

Currency	$-2\ln\lambda$-Value	χ^2-Benchmark	Statement
USD/BTC	342.780	3.841	Reject
USD/LTC	$0.05 \cdot 10^1 2$	3.841	Accept
USD/ETH	$0.07 \cdot 10^1 2$	3.841	Accept
USD/XRP	$0.09 \cdot 10^1 2$	3.841	Accept

Likelihood ratio test results for virtual intra-day data. Table notes: Degree of freedom $k = 1$. Since the Subbotion distribution equals a Laplace distribution for $\kappa = 1$, it comes as no surprise that the Likelihood ratio test yields better results because the κ parameters for the intra-day data reported in S8 Table are close to unity. This leads to the impression that the Subbotin distribution fits the intra-day data better, as the test statistics displayed in S8 Table are also lower, however not significant.
