# Supplementary material for: What’s left after the hype? An empirical approach comparing the distributional properties of traditional and virtual currency exchange rates
Source: PLoS One. 2019 Jul 26;14(7):e0220070. doi: 10.1371/journal.pone.0220070 (PMC6660129; doi:10.1371/journal.pone.0220070)
Supplement: S11 Table — (PDF) [file pone.0220070.s023.pdf]

S11 Table.

	**Ljung-Box**		**Box-Pierce**	
	Statistic	P-Value	Statistic	P-Value
USD/BTC	208.251	$6.15 \cdot 10^-40$*	208.089	$6.66 \cdot 10^-40$*
USD/LTC	188.155	$1.00 \cdot 10^-35$*	188.045	$1.05 \cdot 10^-35$*
USD/ETH	158.326	$1.65 \cdot 10^-29$*	158.190	$1.76 \cdot 10^-29$*
USD/XRP	201.597	$1.53 \cdot 10^-38$*	201.481	$1.62 \cdot 10^-38$*

Ljung-Box and Box-Pierce test results for virtual intra-day data. Table notes: The null hypothesis that data are uncorrelated to lag 8 is or is not rejected at the 5 percent level. *: These tests are rejected at the 5 percent level.
